# Transcription Analysis of the Myometrium of Labouring and Non-Labouring Women

**DOI:** 10.1371/journal.pone.0155413

**Published:** 2016-05-13

**Authors:** Gemma C. Sharp, James L. Hutchinson, Nanette Hibbert, Tom C. Freeman, Philippa T. K. Saunders, Jane E. Norman

**Affiliations:** 1 Tommy’s Centre for Maternal and Fetal Health and Medical Research Council (MRC) Centre for Reproductive Health, University of Edinburgh, Edinburgh, United Kingdom; 2 Systems Immunology Group, Division of Genetics and Genomics, The Roslin Institute and Royal (Dick) School of Veterinary Studies, University of Edinburgh, Edinburgh, United Kingdom; Shanghai Jiaotong University School of Medicine, CHINA

## Abstract

An incomplete understanding of the molecular mechanisms that initiate normal human labour at term seriously hampers the development of effective ways to predict, prevent and treat disorders such as preterm labour. Appropriate analysis of large microarray experiments that compare gene expression in non-labouring and labouring gestational tissues is necessary to help bridge these gaps in our knowledge. In this work, gene expression in 48 (22 labouring, 26 non-labouring) lower-segment myometrial samples collected at Caesarean section were analysed using Illumina HT-12 v4.0 BeadChips. Normalised data were compared between labouring and non-labouring groups using traditional statistical methods and a novel network graph approach. We sought technical validation with quantitative real-time PCR, and biological replication through inverse variance-weighted meta-analysis with published microarray data. We have extended the list of genes suggested to be associated with labour: Compared to non-labouring samples, labouring samples showed apparent higher expression at 960 probes (949 genes) and apparent lower expression at 801 probes (789 genes) (absolute fold change ≥1.2, rank product percentage of false positive value (RP-PFP) <0.05). Although half of the women in the labouring group had received pharmaceutical treatment to induce or augment labour, sensitivity analysis suggested that this did not confound our results. In agreement with previous studies, functional analysis suggested that labour was characterised by an increase in the expression of inflammatory genes and network analysis suggested a strong neutrophil signature. Our analysis also suggested that labour is characterised by a decrease in the expression of muscle-specific processes, which has not been explicitly discussed previously. We validated these findings through the first formal meta-analysis of raw data from previous experiments and we hypothesise that this represents a change in the composition of myometrial tissue at labour. Further work will be necessary to reveal whether these results are solely due to leukocyte infiltration into the myometrium as a mechanism initiating labour, or in addition whether they also represent gene changes in the myocytes themselves. We have made all our data available at www.ebi.ac.uk/arrayexpress/ (accession number E-MTAB-3136) to facilitate progression of this work.

## Introduction

The molecular mechanisms that initiate normal human labour at term are still not fully understood. Although research suggests many fetal, maternal and placental inflammatory and endocrine factors play a role, the relative importance of each of these and/or their interactions is unclear [1]. These gaps in our understanding seriously hamper the development of effective ways to predict, prevent and treat spontaneous preterm labour (PTL) [2]. Given that preterm birth is the single biggest cause of neonatal mortality and morbidity [3,4], this lack of understanding is an important issue. Around 45% of preterm births are preceded by PTL. It is increasingly clear that PTL is a complex syndrome composed of multiple mechanisms [4]. PTL might be caused by early activation of the normal mechanisms that initiate labour, or pathological insults might trigger PTL through different mechanisms. PTL is difficult to predict using known risk factors such as intrauterine infection, previous preterm birth and maternal smoking [4–6], and biomarkers such as cervical length and cervicovaginal levels of fetal fibronectin [7,8]. PTL is also difficult to treat once it has begun and current strategies do not substantially improve neonatal or maternal outcomes [9,10]. Crucially, existing knowledge has also not led to a substantial improvement in clinical outcomes for women presenting in established PTL, although prevention of preterm birth with progesterone shows promise [11]. Clearly there are still large gaps in our knowledge, particularly regarding the complex interactions that may exist between the molecular pathways identified so far. A better understanding of the molecular mechanisms of normal labour at term will help inform studies of preterm labour.

Over the past 15 years, several research groups have published microarray data comparing gene expression in labouring and non-labouring myometrium [12–21]. The myometrium is the tissue that contracts to expel the baby, so it is arguably the most relevant tissue in which to conduct such studies. Although there is some agreement between microarray studies, a clear labour-associated myometrial gene expression signature has not been identified. This may be partly due to the small number of samples used in previous studies—a problem that often arises because human myometrial tissue is relatively inaccessible to study during pregnancy and because researchers select samples from women with similar base characteristics (such as gestational age, parity, BMI, maternal age and indication for Caesarean delivery) to reduce non labour-associated variation between samples. Another possible reason that a clear labour-associated myometrial gene expression signature has not yet been identified is that each study uses different microarray platforms, researchers, locations and analysis methods and the data not analysed optimally.

Including more samples and meta-analysing microarray data gathered from different studies can improve statistical power to detect differentially expressed genes. When studies were subjected to a meta-analysis, differences between studies (heterogeneity) can be assessed and studies with higher statistical power have a greater impact on summary statistics. In addition to traditional statistical approaches, a network clustering approach may also be useful in the identification of genes and biological processes associated with labour. Such techniques consider higher-order interactions between genes and/or samples and are particularly useful when applied to noisy datasets because spurious relationships tend not to influence the shape of the resultant network graph [22].

In this paper, we describe the results of the largest-to-date microarray experiment to compare gene expression in labouring and non-labouring myometrium. We combined statistical and network clustering approaches to identify genes and biological processes associated with labour. We also perform the first formal meta-analysis on publically-available sample level data from similar published microarray experiments. We believe that these approaches will lead to more accurate and generalizable conclusions that may be useful in helping to understand molecular mechanisms in normal labour at term, and inform studies of preterm labour.

## Methods

### Myometrial tissue samples

Biopsy samples of full thickness lower segment specimens of human myometrium were selected from the Edinburgh Reproductive Tissue Biobank (ERTBB, http://www.crh.ed.ac.uk/biobank/). All tissue samples were collected during Caesarean section from participant tissue donors with informed and written consent, according to the ethical approval and governance granted to the Edinburgh Reproductive Tissues BioBank by the West of Scotland Research Ethics Committee 4 (09/S0704/3). In order to maximise biological replicates on the array, all 22 labouring samples that fulfilled our inclusion criteria and that were stored in the ERTBB as of August 2012 were selected. In order to minimise inter-group variation in baseline characteristics, a second group of non-labouring samples (n = 26) were selected by matching to labouring samples on maternal BMI, gestational age, parity and maternal age. We did not exclude smokers (data not available) or women who had received medication to induce labour (n = 11). We restricted the number of non-labouring samples to 26 so that all samples (n = 48) could fit on four array chips.

Forty-five of the samples had been transferred into RNA*later*® (Life Technologies, Invitrogen, Carlsbad, CA, USA) immediately after collection and then stored at -80°C. Three labouring samples had not been transferred to RNA*later*® before freezing but were also included in the array analysis in order to maximise the number of labouring samples. We noted that a previous microarray experiment has shown that there is no significant difference in quantitative RNA expression in fresh or frozen myometrial tissue that has or has not been stored in RNA*later*® [23].

### RNA preparation

Total RNA was isolated using TRI Reagent® and the Qiagen RNeasy Lipid Tissue kit protocol (Qiagen, Valencia, CA, USA). The quantity and quality of RNA was assessed using a NanoDrop 1000 (Thermo Scientific, Wilmington, DE, USA). RNA quality was further assessed in the biotin-labelled samples by the Wellcome Trust Clinical Research Facility, Edinburgh using a Bioanalyzer 2100 (Agilent Technologies, Wilmington, DE, USA). To prepare samples for the microarray experiment, 450ng total RNA was amplified and biotin-labelled using the Illumina® TotalPrep^TM^ RNA Amplification Kit (Ambion, Austin, TX, USA).

### Illumina HT-12 v4.0 BeadChip Expression microarray

Samples were split randomly over four Illumina HT-12 v4.0 BeadChips to minimise any effect of inter-chip variability. One sample was used per well. The chips were imaged using a BeadArray Reader and raw data were obtained with Illumina BeadStudio software. Raw and processed data are available at www.ebi.ac.uk/arrayexpress/ under accession number E-MTAB-3136.

#### Data preprocessing

Raw data were preprocessed using the Lumi package[24–27] in R version 2.14.1. The data were subjected to Robust Multichip Average (RMA) background correction before quantile normalisation to remove non-biological systematic variation [28]. Probes were also annotated with gene name, official gene symbol, Unigene ID, Entrez gene ID, and Gene Ontology (GO) terms according to the Illumina probe ID. Where there were genes with mean expression values of less than 100 (rounded from the median expression value for negative control probes) for both labouring and non-labouring groups, we assumed that the genes were not expressed in the samples. Data for these genes were therefore removed from further analyses.

#### Statistical analysis

For each probe, the R package Limma [29] was used to calculate the fold-change in mean expression between groups and the significance of differential expression using a non-parametric Rank Product [30] approach. The Rank Product approach reduces the chance of false negative results. It is corrected for multiple testing using the percentage of false positives (PFP) and reported as an RP-PFP value. This value may be considered as roughly equivalent to a P-value, so genes with an RP-PFP ≤0.05 and fold change (effect size) >1.2 or <-1.2 were considered differentially expressed and taken forward for further analysis.

#### Network graph analysis

Using expression data for the top up- and down-regulated genes, we created sample-sample and probe-probe network graphs in Biolayout *Express*^3D^ [31]. In the sample-sample graph, each ‘node’ (sphere) represents a sample connected to others by ‘edges’ weighted according to the strength of the sample-sample Pearson’s correlation coefficient. All correlation values (*r*) above 0.85 were used to draw a graph of this similarity matrix. Samples with similar gene expression signatures appear closer (connected) to each other in the graph, thus creating local structure within the graph. This structure represents overall similarity between samples, which can be used to explore correlations with clinical differences such as membership of the labouring/non-labouring group, body mass index (BMI) category, nulliparous/multiparous, etc. The Markov Clustering algorithm (MCL) [32,33] was performed with the inflation value (MCLi) set at 3.0 to give an unbiased assessment of how the samples cluster. Cluster membership was compared to labour status using a Fisher’s exact or chi-squared test.

In the probe-probe graph, each node represented a probe connected to others by edges weighted according to the similarity (above a threshold of *r* = 0.80) of expression profile, that is, the degree of co-regulation in all samples. MCL clustering (MCLi = 2.2) was performed to give an unbiased assessment of how the probes cluster. Average (mean) expression profiles for each cluster were examined.

#### Functional analysis

Lists of official gene symbols representing 1) down-regulated genes (fold change <-1.2 and RP-PFP ≤0.05) and 2) up-regulated genes (fold change >1.2 and RP-PFP ≤0.05) were uploaded to DAVID (The Database for Annotation, Visualization and Integrated Discovery [34,35]). The lists were assessed for biological process GO (Gene Ontology [36]) term enrichment and KEGG (Kyoto Encyclopaedia of Genes and Genomes [37,38]) pathway enrichment. We also looked up certain genes in the primary cell atlas [39] on the BioGPS website [40] to explore average expression in different human tissues.

### Validation with qRT-PCR

qRT-PCR gene expression assays were chosen to validate some of the specific differences identified in the microarray (assay IDs and the reasons for choosing specific genes are given in Table A in S5 File). Quantitative RT-PCR was performed using 45 (20 labouring, 25 non-labouring) of the original RNA samples used in the microarray experiment. By the time that qRT-PCR was performed, three of the original samples had degraded in storage (as assessed by the 260nm/280nm ratio using a NanoDrop 1000 (Thermo Scientific, Wilmington, DE, USA)) and no longer gave adequate quality readings, therefore these samples were excluded from subsequent PCR analysis. Gene expression was determined using the standard curve method, relative to the reference gene 18S. Pre-designed, inventoried TaqMan gene expression assays (Applied Biosystems (Life Technologies), Foster City, CA, USA) were used to measure the expression of ten genes (*RBM42*, *SHROOM4*, *FABP4*, *IGFBP5*, *MYH11*, *TPM1*, *IL6*, *IL8*, *MT1E*, *OXTR*). qRT-PCR was performed using an ABI 7900HT (Applied Biosystems, Carlsbad, US), on 384 well plates. T-tests were used to analyse whether the delta CT values (raw CT value for the target gene minus the raw CT value for 18S) were significantly different in labouring and non-labouring samples. We also assessed the suitability of 18S as a reference gene by examining the mean raw CT values in the labouring and non-labouring groups.

### Replication using microarray meta-analysis

In addition to the wet-lab validation of the microarray, we performed a meta-analysis of data derived from similar microarray studies, including the current study. Further details of the meta-analysis methods are provided in S1 File. Briefly, we searched the literature for eligible studies, obtained raw or normalised expression data, pre-processed the data consistently, compared lists of genes showing differential expression according to labour status and performed fixed effects inverse variance-weighted meta-analysis to compare study-specific standardised mean differences for certain genes of interest.

## Results

### RNA quality

Before biotin labelling, RNA samples had a mean 260nm/280nm ratio of 2.07 (standard deviation 0.04), confirming their quality. Fig A in S5 File shows boxplots and intensity plots to visualise the distribution of microarray expression values across all probes and all samples before and after quantile normalisation. There were no large variations in expression values between samples and all data were included for further analysis.

### Statistical analysis of sample characteristics

Table 1 summarises the characteristics of women in the labouring (n = 22) and non-labouring (n = 26) groups (more details are provided in Table B in S5 File). Although within-group variance was reasonably high (as expected in these unselected samples), there were no between-group differences in mean/median values of parity, maternal age, maternal BMI or gestational age at delivery, indicating that the groups are well-matched. Four women in the labouring group and two women in the non-labouring group delivered preterm before 37 weeks (earliest gestations: labouring group, 33+4 weeks; non-labouring group, 31+2 weeks). Half of the women in the labouring group had received prostaglandins (n = 3), oxytocin (n = 7) or both treatments (n = 1) to induce or aid the progression of labour. Fold-changes generated in a sensitivity analysis omitting these women were consistent with those generated in the main analysis (median absolute difference: 0.005; interquartile range -0.02, 0.03; correlation = 0.84; direction of effect was the same for 90.1% of probes). We assume, therefore, that any difference in gene expression between the non-labouring and labouring groups was likely to be primarily the result of labour status rather than variation in any of the other clinical characteristics measured.

**Table 1:** Summary of characteristics of women in the labouring and non-labouring groups.

		Labouring group	Non-labouring group	P-value*
Tissue storage (n)	RNA later	19	26	n/a
	No RNA later	3	0	n/a
Indication for Caesarean section (n)	Fetal distress	3	0	n/a
	Failure to progress	11	0	n/a
	Breech presentation	3	11	n/a
	Obstetric history	0	6	n/a
	Other or missing	5	9	n/a
Mean maternal age (95% CI)		32 (29.1–34.4)	32 (30.8–34.6)	0.52
Mean body mass index (kg/m^2^) (95% CI)		28.0 (24.4–31.6)	28.4 (25.3–31.6)	0.86
Method of induction (n)	Prostaglandins	3	0	n/a
	Oxytocin	7	0	n/a
	Prostaglandins and oxytocin	1	0	n/a
	None	11	26	n/a
Mean gestation at delivery (weeks) (95%CI)		40 (39–41)	39 (38–40)	0.15
Preterm (n)		2	4	n/a
Median parity (weeks; interquartile range)		0 (0–1)	0 (0–1)	0.45
Mean cervical dilation (cm) (95% CI)		5.8 (4.3–7.3)	n/a	n/a

* P-values were calculated using t-tests.

### Statistical analysis of differential expression

**Fig 1:**
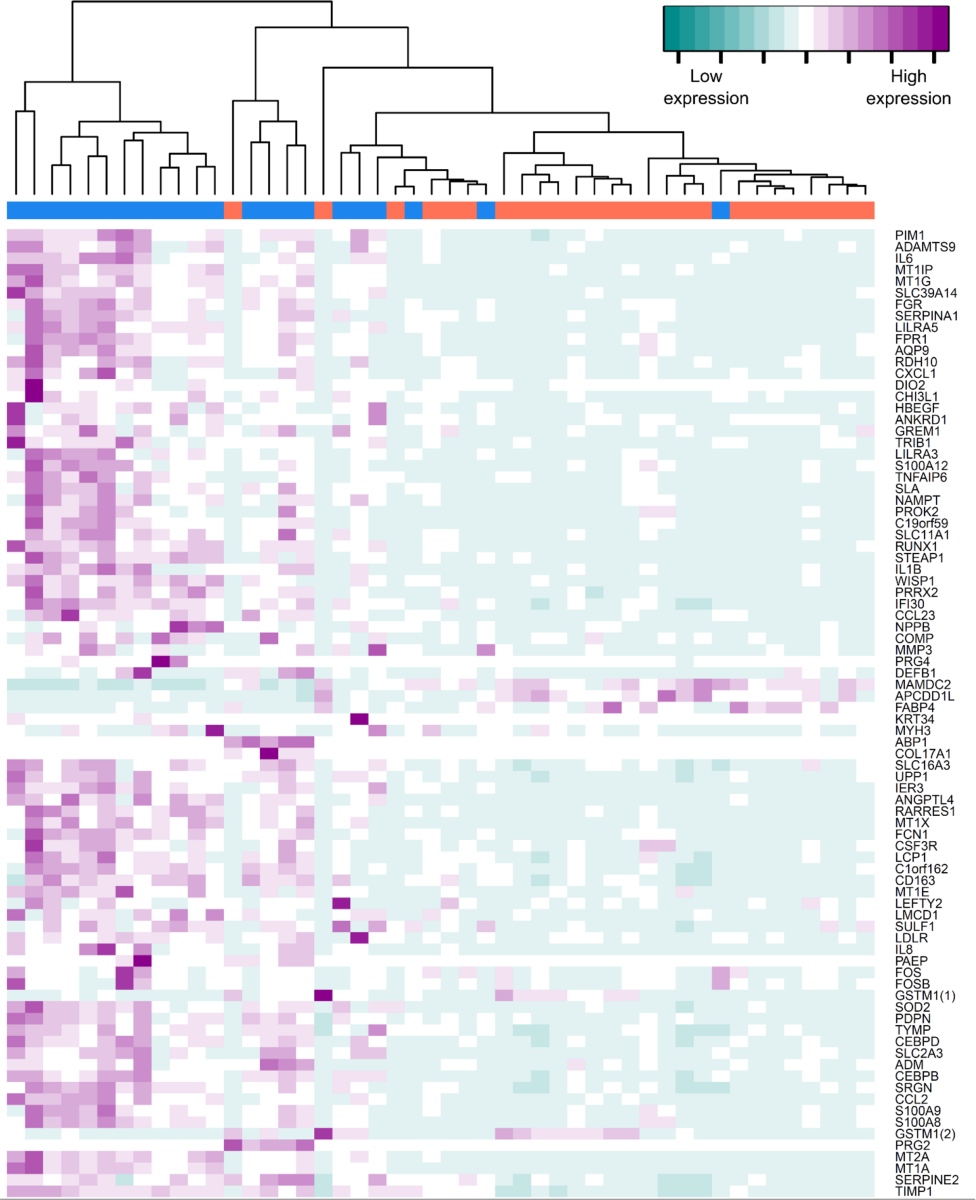
A heatmap to show how genes and samples cluster based on similar expression levels. The bar at the top indicates the sample group (pink = labouring, blue = non-labouring). Normalised standardised expression values are indicated on a colour scale with purple indicating high expression and cyan indicating low expression. The heatmap was created using genes with a non-labour to labour fold change of >2 or <-2 and an RP-PFP ≤0.0001.

A complete list of differentially expressed genes with an RP-PFP value <0.05 and an absolute fold change ≥1.2 is presented in S2 File. According to these criteria, 960 probes (949 genes) showed higher expression and 801 probes (789 genes) showed lower expression in labouring samples compared to non-labouring samples. Fig 1 shows a heatmap of genes with an absolute fold-change >2 and an RP-PFP of ≤0.0001. Hierarchical clustering demonstrates that samples tend to cluster according to labour status.

### Functional analysis

Table C and D in S5 File present the top five clusters of GO terms for biological processes associated with the genes that showed higher and lower expression in labour, respectively. According to this functional analysis, biological processes that are active in labour are those mainly associated with inflammation, cell movement/migration, response to hormone stimulus, regulation of cell death and angiogenesis. Biological processes that are more active in the non-labouring myometrium are mainly associated with responses to hormones, muscle development, cytoskeleton organisation, ion homeostasis, neuron development and cell adhesion. KEGG pathways active in labour include NOD-like receptor signalling, MAPK activation and cytokine-cytokine receptor signalling. These are all important inflammatory pathways. Interestingly pathways less active in labour include vascular smooth muscle contraction and calcium signalling. However, rather than a change in transcription within myometrial muscle cells, this may represent a change in the composition (effectively dilution in quantity of muscle cells) of the myometrial tissue due to leukocyte infiltration at labour[41,42] or possibly the presence of more blood in labouring samples.

### Network graph analysis

In addition to the more traditional statistical analyses described above, we also used a network graph approach. MCL clustering of these network graphs allowed us to understand the relationships between samples, genes and labour status at a finer level.

#### Sample-sample associations

**Fig 2:**
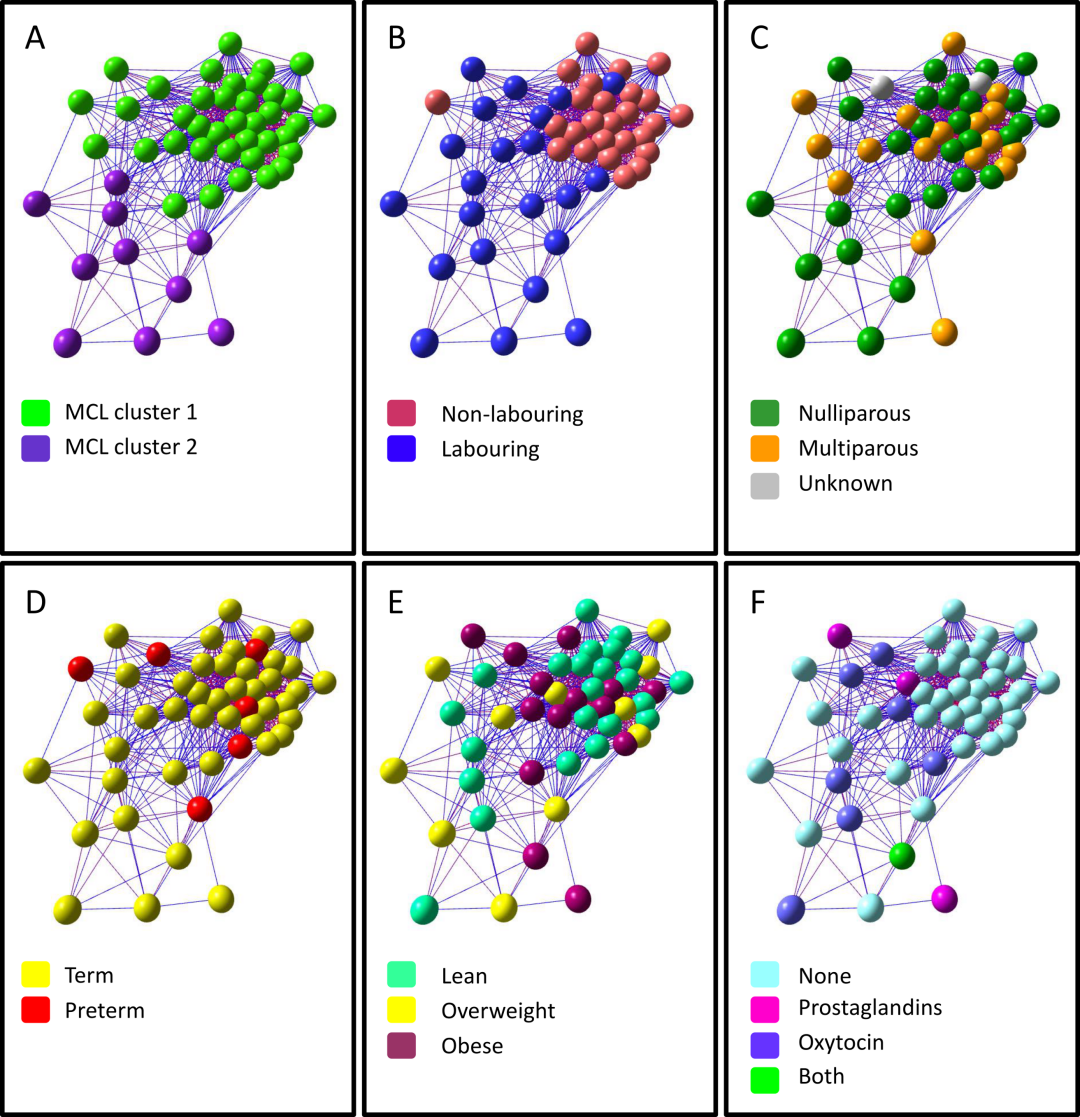
Sample-sample network graph, in which each node represents a different sample. Edges are coloured to reflect the Pearson correlation that they represent. Red edges represent a high correlation and blue edges represent a low correlation. The same graph is coloured by A. unbiased MCL cluster number, B. labour status, C. parity, D. gestational age at delivery, E. body mass index (BMI), and F. pharmaceutical treatment.

The sample-sample network graph shown in Fig 2 was built using expression data for the top up- and down-regulated genes (differentially expressed genes with a RP-PFP value <0.05 and an absolute fold change ≥1.2). MCL clustering (MCLi = 3.1) of the graph’s structure highlighted two clusters (Fig 2A). When nodes are coloured according to labour status (Fig 2B) it is clear that MCL cluster 1 (38 samples) contains all 26 of the non-labouring samples (with 12 labouring samples included) and MCL cluster 2 (10 samples) contains labouring samples only (Fisher’s exact test P-value = 0.0005). This suggests that the samples cluster by labour status, and the appearance of labouring samples in both clusters suggests that labouring samples show more variation than non-labouring samples (an observation born out at the gene expression level–see below). Colouring the graph by parity (Fig 2C), gestational age at delivery (Fig 2D) and BMI (Fig 2E) shows that nodes do not tend to cluster according to these other characteristics (Fisher’s exact test P-value for parity [nulliparous versus multiparous]: 0.88; for gestational age [preterm versus term]: 1; for BMI [overweight/obese versus normal weight]: 0.84). Fig 2F shows the graph coloured according to pharmaceutical treatment the woman had received to induce/augment labour. No non-labouring women received any treatment and, in agreement with the sensitivity analysis described above, samples do not cluster according to pharmaceutical treatment within the labouring group (Fisher’s exact test P-value [any treatment versus no treatment]: 0.06). Samples also do not cluster by maternal age (Fisher’s exact test P-value [under 36 years versus over 36 years]: 0.05).

### Co-expression analysis

**Fig 3:**
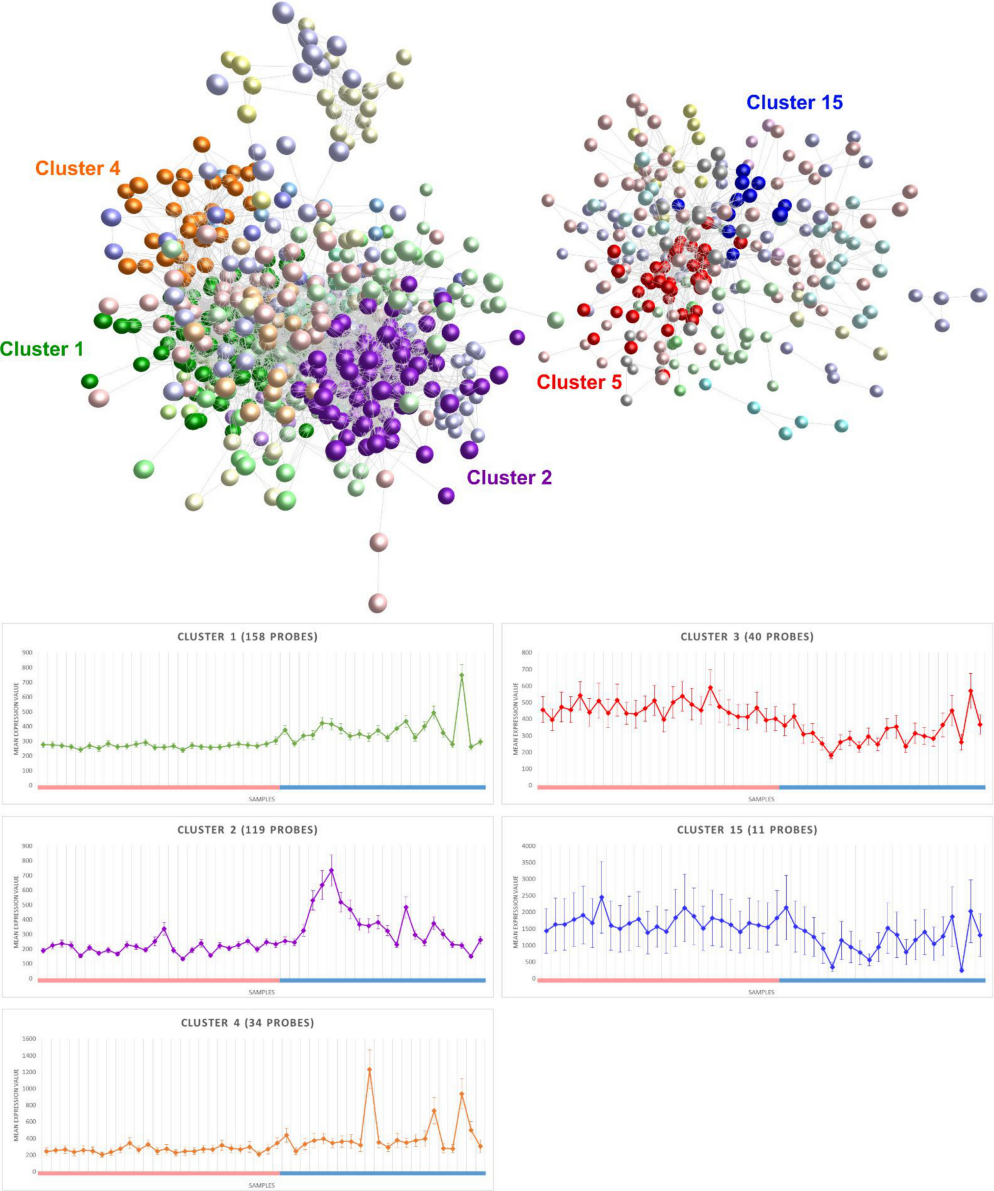
Probe-probe network graph, in which each node represents a different probe. Nodes are coloured according to membership of different MCL clusters. The graphs show the mean expression profiles of clusters 1,2,3,4 and 15. Samples are plotted on the x-axes: non-labouring samples are represented by the pink bar and labouring samples are represented by the blue bar. Error bars indicate standard errors.

In a network graph where each node represents data from a differently expressed transcript (Fig 3), after applying a correlation threshold of *r* = 0.8, there were 1,209 nodes connected by 11,030 edges. The graph forms two main components. The larger component is composed of genes that showed higher expression in labouring compared to non-labouring samples and the smaller component is composed of genes that showed lower expression in labouring compared to non-labouring samples. MCL clustering identified 85 distinct clusters of transcripts that showed similar expression profiles (co-regulation across samples). Average (mean) expression profiles for interesting clusters are shown in the graphs in Fig 3. Cluster 1 contained 158 transcripts that showed higher expression in labouring samples than non-labouring samples. The genes are mostly involved in RNA and protein processing (S3 File), which indicates that labour is associated with increased transcription and translation in myocytes, leukocytes or both. Of particular interest in this cluster is IL24, which shows very high expression in several labouring samples and almost no expression in any of the non-labouring samples (Fig B in S5 File). Similarly, Cluster 4 is comprised of 33 genes that show higher expression in labouring samples and mostly represent early response genes such as *ZFP36*, *EGR1* and *ATF3*. Again, this suggests that labour is an active process associated with increased gene expression. The 119 genes in Cluster 2 also show higher expression in labouring samples. These genes (S3 File) are mostly involved in inflammatory responses, chemotaxis and cell activation, suggestive of an infiltration of leukocytes into the myometrial tissue at labour. High expression of genes that are usually highly expressed in neutrophils such as *CXCL2*, *CD53*, *CD48*, *NCF2*, *NCF4* and *LRG1* (S4 File) suggests that these leukocytes are likely to be largely made up of neutrophils. Cluster 3 contains 40 genes that show lower expression in labouring samples than non-labouring samples. These genes include *AHNAK*, *AHNAK2*, *RAB11FIP2*, *PLCL1*, *SCARA3* and *TMEM123*, which are all usually highly expressed in the uterus according to Unigene ESTs counts (http://www.ncbi.nlm.nih.gov/unigene/). The lower expression of these genes in labouring samples could be due to a decrease in transcription in the myocytes but a more plausible explanation is that labouring samples are composed of fewer uterine smooth muscle cells than non-labouring samples i.e. that the composition of the myometrium changes at labour and uterine smooth muscle cells are diluted. Similarly, classic markers of muscle tissue *MYH11*, *MYL9* and *TAGLN* are all in Cluster 15, which is a cluster of genes that showed lower expression in labour.

### Validation with qRT-PCR

For qRT-PCR data analysis, all genes were analysed relative to expression of 18S, which was an appropriate reference gene because it was not differentially expressed between non-labouring and labouring groups (mean CT value plus 95% confidence intervals of labouring group: 11.8 (9.5–14.1), mean CT value plus 95% confidence intervals of non-labouring group: 10.8 (9.2 to 12.4). Log2 ratios of expression in non-labouring and labouring samples calculated using microarray data were highly correlated with those calculated using qRT-PCR (Pearson’s correlation coefficient *r* = 0.99; Fig C in S5 File). Both technologies found that Interleukin-8 (*IL8*), Interleukin-6 (*IL6*) and Metallothionein 1E (*MT1E*) showed higher expression in labour, Oxytocin receptor (*OXTR*), Myosin heavy chain 11 (*MYH11*) and Fatty acid binding protein 4 (*FABP4*) showed lower expression in labour and RNA binding motif protein 42 (*RBM42*) and Shroom family member 4 (*SHROOM4*) were not differentially expressed between groups. Although microarray and qRT-PCR both found that Insulin-like growth factor binding protein 5 (*IGFBP5*) showed lower expression at labour, this did not reach statistical significance at P = 0.05 according to qRT-PCR. Microarray and qRT-PCR results were inconsistent at Tropomyosin 1 (*TPM1*) (Fig C in S5 File).

### Replication using meta-analysis of published data

In addition to technical validation of our results using qRT-PCR on the same samples as those used for the microarray, we were able to replicate some of our results in different samples through a formal meta-analysis of published data.

The ten eligible studies identified through a literature search for myometrial microarrays are outlined in Table E in S5 File. Unfortunately, raw data were available for only two: Bukowski (2006) [14] and Weiner (2010) [15]. These studies were included in a meta-analysis, along with the microarray study conducted in this paper (referred to as Sharp). This meta-analysis used 25 samples (13 labouring, 12 non-labouring), plus the 48 used in Sharp, giving a total of 73 samples (35 labouring, 38 non-labouring).

Each study has a different complement of probes, but 4396 genes were covered on the array platforms of all three studies. Of these, the number of significantly (RP-PFP<0.05) differentially expressed (absolute fold change of >1.2) genes in Sharp, Weiner and Bukowski, was 1330 (1099 unique), 576 (469 unique) and 192 (192 unique), respectively. 64.4% of the genes identified in Weiner and 31.8% of the genes identified in Bukowski were also identified in Sharp with the same direction of effect. 24.0% of the genes identified in Bukowski were also identified in Weiner with the same direction of effect. Studies did not agree on the direction of effect at 1.3%, 8.9% and 6.3% of identified genes for comparisons between Weiner and Sharp, Bukowski and Sharp and Weiner and Bukowski, respectively. Only 34 genes were identified as differentially expressed in the same direction in all three studies (Table 2). This low concordance is partly because lower sample sizes in Weiner and Bukowski mean they have a lower statistical power to detect differential expression. Accordingly, when just considering direction of effect and not applying P-value or effect size thresholds we see much higher concordance rates: Of the 1099 unique genes identified as significantly differentially expressed in Sharp, 94.4% and 64.7% showed the same direction of effect in Weiner and Bukowski, respectively. However, it is clear that the concordance between Sharp and Weiner is higher than the concordance between either of those studies with Bukowski. For example, at the most differentially expressed gene in Sharp (IL8, fold change 9.6), Weiner finds a large fold change in the same direction (24.9) but Bukowski finds a smaller fold change in the inverse direction (-1.4).

**Table 2:** Fold changes for genes agreed to be significantly differentially expressed in the same direction by each meta-analysed study.

Gene ID	Gene name	Sharp	Weiner	Bukowski
CCL2	Chemokine (C-C motif) ligand 2	4.01	3.41	1.32
S100A8	S100 calcium binding protein A8	3.33	3.58	1.27
PRG2	Proteoglycan 2	2.14	1.62	4.24
SPP1	Secreted phosphoprotein 1	2.00	1.88	1.71
PLA2G2A	Phospholipase A2, group IIA (platelets, synovial fluid)	1.64	2.71	1.49
COL1A2	Collagen, type I, alpha 2	-1.2	-1.48	-1.22
PRSS23	Protease, serine, 23	-1.21	-1.61	-1.36
TMEM59	Transmembrane protein 59	-1.21	-1.42	-1.82
AP3S1	Adaptor-related protein complex 3, sigma 1 subunit	-1.24	-1.26	-1.91
ATP2B4	Atpase, Ca++ transporting, plasma membrane 4	-1.24	-1.44	-1.70
GAS1	Growth arrest-specific 1	-1.26	-1.42	-1.39
ISCU	Iron-sulfur cluster scaffold homolog	-1.26	-1.23	-1.67
LAMA2	Laminin, alpha 2	-1.26	-1.65	-1.87
PRKAR1A	Protein kinase, camp-dependent, regulatory, type I, alpha	-1.28	-1.30	-2.27
CAV1	Caveolin 1, caveolae protein, 22kda	-1.29	-1.28	-1.45
AHNAK2	AHNAK nucleoprotein 2	-1.32	-1.55	-1.54
MFAP5	Microfibrillar associated protein 5	-1.32	-1.44	-2.05
RNASE1	Ribonuclease, rnase A family, 1 (pancreatic)	-1.32	-1.25	-1.35
TMEM123	Transmembrane protein 123	-1.34	-1.22	-1.96
ALDH1A2	Aldehyde dehydrogenase 1 family, member A2	-1.35	-1.88	-1.49
METTL7A	Methyltransferase like 7A	-1.4	-1.57	-2.26
RASSF2	Ras association (ralgds/AF-6) domain family member 2	-1.41	-1.58	-2.00
CALD1	Caldesmon 1	-1.44	-1.32	-1.5
FHL1	Four and a half LIM domains 1	-1.44	-1.46	-1.34
TUBB2A	Tubulin, beta 2A	-1.44	-1.23	-1.6
VCL	Vinculin	-1.44	-1.22	-1.52
PARVA	Parvin, alpha	-1.46	-1.31	-1.23
TPM1	Tropomyosin 1 (alpha)	-1.47	-1.22	-1.20
TCEAL1	Transcription elongation factor A (SII)-like 1	-1.51	-1.44	-1.63
NR2F2	Nuclear receptor subfamily 2, group F, member 2	-1.52	-1.46	-1.87
SYNM	Synemin, intermediate filament protein	-1.52	-1.32	-1.89
DAAM1	Dishevelled associated activator of morphogenesis 1	-1.54	-1.86	-2.80
TCEAL4	Transcription elongation factor A (SII)-like 4	-1.63	-1.38	-1.28
SVIL	Supervillin	-1.86	-1.38	-1.85

**Fig 4:**
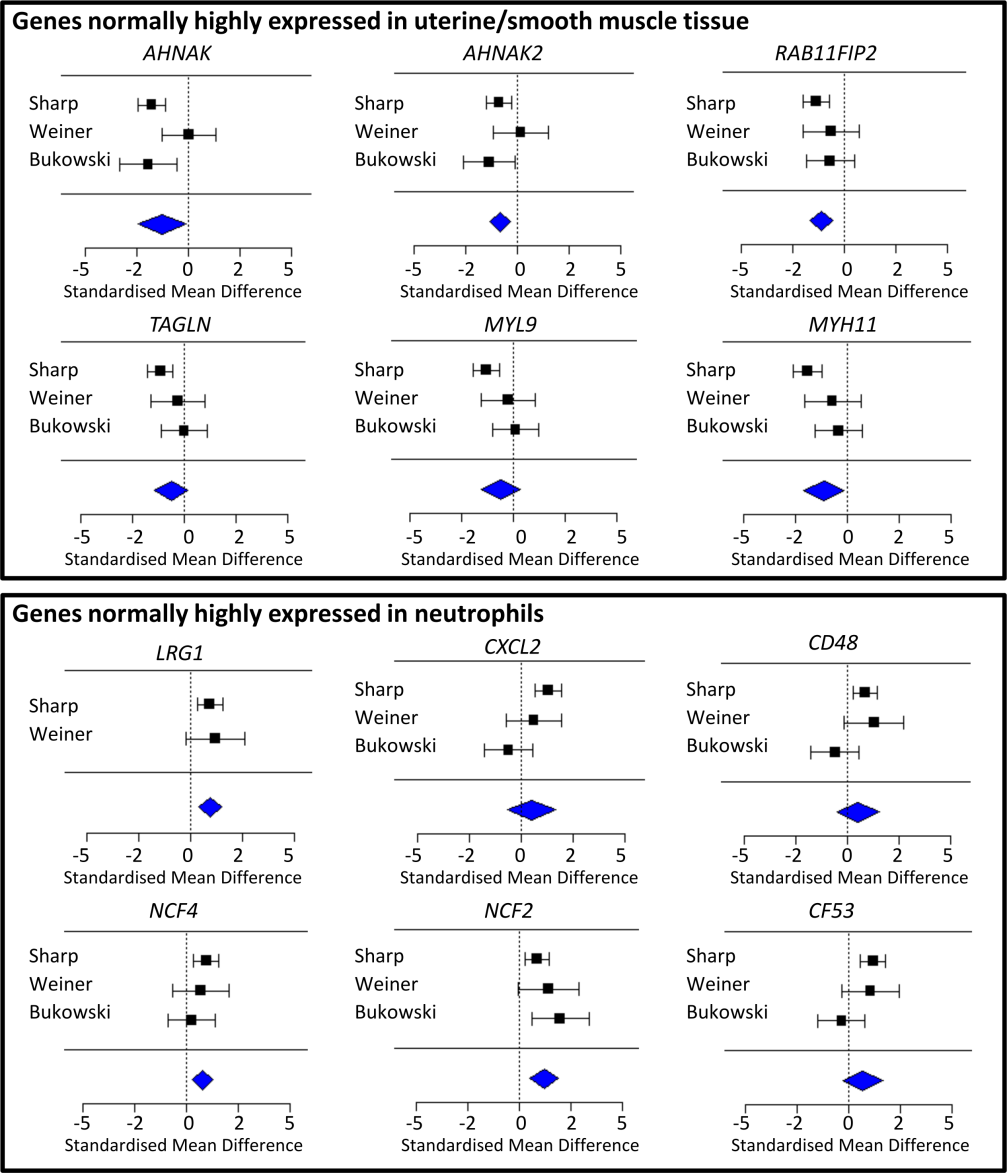
Forest plots to illustrate the standardised mean difference between labouring and non-labouring groups in Sharp, Weiner and Bukowski. Summary statistics, indicated by the blue diamond, were calculated via inverse variance weighted meta-analysis. The array platform used by Bukowski did not cover all selected genes, so Bukowski could not be included in all meta-analyses.

The results of a formal random-effects inverse-variance-weighted meta-analysis on the expression of neutrophil and smooth muscle myofilament markers [43] in each study are shown in Fig 4. In general, studies agreed on the direction of the changes and heterogeneity (which was assessed using Tau^2^ and significance of the Chi^2^ statistic) was low (Table F in S5 File). This suggests that, in all three studies, there is evidence that labouring samples contain more neutrophils and relatively fewer smooth muscle cells than non-labouring samples. Fig D in S5 File shows the results of a meta-analysis on the expression of the ten genes that were selected for validation by qRT-PCR. All studies agree on the direction of the change in expression between non-labouring and labouring samples, except for at *IL6*, *IL8* and *MT1E*, where Sharp and Weiner agree that these genes show higher expression at labour, but Bukowski does not. Interestingly, although the differential expression of *TPM1* seen in the Sharp microarray data could not be validated with qRT-PCR, the Sharp, Weiner and Bukowski microarray data all agree that *TPM1* shows lower expression in labouring compared to non-labouring samples.

## Discussion

In our array analysis, which is over 20% larger in terms of sample size than any of those in the published literature, the myometrial transcriptome of labouring women differed considerably from that of non-labouring women. 1,761 genes were differentially expressed between the non-labouring and labouring groups, which is considerably more than identified by previous microarray experiments of this kind which have been less powered to detect differential expression due to using smaller sample sizes and have used smaller array platforms with less coverage of the transcriptome [15,17]. Network graph analysis showed that in terms of overall gene expression, non-labouring samples were very similar to each other and in general different from labouring samples. This suggests that non-labouring women have a similar myometrial gene expression profile to each other, but this changes at labour, and variation between labouring women might be explained by variation in the stage of labour. We did not attempt to formally validate this hypothesis in our study, given a lack of consensus as to how labour progress should be measured. It is worth noting that there was considerable heterogeneity amongst the labouring samples with some not differing greatly from the non-labouring samples, whilst others show considerable differences. This heterogeneity undermines a purely statistical approach to the analysis of these data as comparisons are performed across the group, thereby reducing the overall fold differences in expression when some labouring samples show little overall change.

Traditional statistical analysis combined with functional analysis suggested that the labouring myometrium expresses genes involved in inflammation, cell movement/migration and pathways such as Nod-like receptor signalling and cytokine-cytokine receptor signalling. Pathways identified here as associated with labour are downstream to pathways to NF-kappa B (NF-κB) activation or involved in cross talk with these pathways. These observations are in agreement with the results of previous human microarray studies [15,17,44–46] and might provide further evidence to support the hypothesis that the initiation of parturition is associated with inflammatory processes. Through the network graph approach we inferred that there was a strong neutrophil expression signature in the labouring samples. A meta-analysis including two previous myometrial microarrays conducted on small numbers of samples showed that neutrophil markers also tended to be more highly expressed in the labouring samples. These findings confirm and extend previous research that has demonstrated (using immunohistochemistry) an influx of leukocytes into the uterine tissue at labour. Studies conducted more than 10 years ago suggested that neutrophils, macrophages and T cells are attracted by increased tissue expression of chemokines and cell adhesion molecules [47,48] to invade the myometrium, cervix and fetal membranes at parturition [41,42], although in our recent mouse study, neutrophil depletion did not avert preterm parturition [49]. The increase in leukocyte density, in part, leads to increased cytokine expression in the myometrium and cervix [41,50]. These cytokines attract yet more leukocytes [51], but also have a pronounced effect on the reproductive tissues, including remodelling of the cervix [52] and stimulating myometrial contraction through prostaglandin synthesis and via direct TLR-4 mediated effects [53–59]. Prostaglandins mediate inflammation and some have a direct contractile effect on myometrial myocytes [60–63]. However, functional analysis suggested that non-labouring samples highly express genes involved in muscle-specific processes and pathways such as muscle development, smooth muscle contraction and calcium signalling. This observation of an apparent down-regulation of muscle-specific processes at labour was surprising and, to our knowledge, has not been discussed explicitly before. However we hypothesise that this may reflect known changes in composition of the myometrial tissue at labour rather than a change in transcription of muscle-specific genes. Indeed, network graph analysis helped us identify that non-muscle specific genes that are usually highly expressed in the uterus were also expressed at lower levels in labouring samples compared to non-labouring samples. Leukocyte infiltration may ‘dilute’ the smooth muscle cell contribution to myometrial tissue gene expression at labour. We initially considered trying to adjust for this dilution effect (for example, by normalising gene expression values to the expression of leukocyte markers) but we did not, as we believe that such an adjustment would be unlikely to be useful or reliable in noisy human data. It is of particular interest that, as we showed in our meta-analysis, the finding of increased neutrophil and decreased muscle cell expression signatures at labour replicates in data from similar microarray experiments and is also in agreement with the results of a recent RNA-Seq experiment [64].

This finding has important implications for the interpretation of similar analyses of gene expression–i.e. it may not be possible to simply say that a gene is ‘up-regulated’ or ‘down-regulated’ in myometrial tissue when in fact transcription of the gene may not change but the transcript may be in relative abundance/scarcity due to differences in tissue composition.

The main strengths of our study are its sample size (the largest to date) and the comprehensive methods used to analyse the data. In studies of parturition, gene expression is likely to show a particularly high degree of variation between individuals. This is not only due to genetic and environmental effects, but also because the time to the onset of spontaneous labour in non-labouring samples is unknown [14]. The large sample size used in this study helps to reduce some of this confounding by increasing the power of the study to detect real changes in gene expression. We selected to maximise the number of samples in the experiment, rather than minimise inter-woman variation. Therefore, there was a high degree of variation within (but not between) each group on BMI, maternal age, gestational age, parity and stage of labour (in the labouring group, assessed by cervical dilation). Although the ideal experimental design would control for these characteristics in this way, perhaps excluding gestational age [15], there is little evidence to suggest that the molecular mechanisms initiating parturition are affected by these factors. Therefore, we believe that prioritising maximal sample number over minimising inter-woman variability is an appropriate strategy. Indeed, MCL clustering of a sample-sample network graph of our data suggested that samples cluster according to labour status but none of the other maternal characteristics on which we had data. We were particularly surprised to find that labouring samples did not cluster according to gestational age, as a previous microarray experiment had suggested that term labour and PTL were driven by different molecular mechanisms [15]. However, only three of our labouring samples were preterm so our results do not necessarily suggest that this is not the case.

This highlights another strength of our study, which is that we combined traditional statistical and novel network graph approaches to offer new insights into variation in gene expression signatures. The network graph approach is more robust to inter-individual heterogeneity and better able to identify broad differences in expression between groups. Additionally, we conducted a formal meta-analysis to study the myometrial transcriptome at labour, which allowed us to compare our results to those of similar microarray studies and further increased the statistical power, reliability and generalizability of results. It was disappointing that of the ten studies eligible for our meta-analysis [12–21], only two [14,15] had made their data available in an online repository. Including more studies with larger sample sizes would have improved the power of our meta-analysis. Nevertheless, this is the first time raw, complete microarray data to study parturition has been formally meta-analysed. Although there is one published meta-analysis of microarray data to study parturition [65], this study, which was published in 2007, did not use raw data. Instead, the authors combined lists of genes reported to be differentially expressed in five human studies of myometrial gene expression in pregnancy, term and preterm labour. The authors found that the remodelling and maturation processes associated with pregnancy last the full course of gestation, but genetic regulation of the onset of parturition was less well characterised. However, use of published gene lists rather than raw microarray data ignores information on genes showing lower levels of differential expression and does not account for the different methods used to generate and analyse the data.

Our own analysis showed that there is low concordance between microarray studies in terms of lists of “significant” genes, even when the data has been pre-processed and analysed in the same way, but this is partly because different array platforms have a different complement of probes. It was more informative to conduct a formal meta-analysis at selected genes using individual sample data. As discussed above, this revealed that studies did agree that labour is associated with higher expression of genes involved in inflammatory processes (particularly neutrophil markers) and lower expression of genes involved in muscle-specific processes. This was also echoed by a recent RNA-Seq experiment that compared gene expression in a small number of non-labouring and labouring women [64]. Overall, this experiment showed high concordance with our own microarray experiment: Of genes identified by the RNA-Seq experiment as showing higher or lower expression in labour compared to non-labour, 51.8% and 57.3% were also identified as showing significantly higher or lower (respectively) expression in our microarray experiment, whereas none were identified as showing significant differential expression in the opposite direction. The list of genes agreed to show higher expression in labour includes *IL6*, *MT1E*, *IL24*, *LRG1* and *CXCL2*, and the list agreed to show lower expression in labour includes *OXTR*, *IGFBP5*, *AHNAK*, *RAB11FIP2*, *TMEM123* and *PLCL1*. This provides further verification that our results replicate using not only different samples but also a different technology [64].

As with all microarray studies of human tissue collected opportunistically, there are some limitations that should be considered when interpreting the results. Firstly, the qRT-PCR validation of the microarray results was conducted on the same samples used in the array. More reliable validation could have been achieved using samples from different women, but this was impossible due to lack of availability of such samples. The large number of samples used in this study means this is less of a limitation than it is for smaller microarray experiments, and we have compared our results to those of published experiments that used different samples. Secondly, the non-labouring samples were collected during elective Caesarean section that was sometimes performed because the woman had previously delivered via Caesarean section due to an underlying uterine pathology. Additionally, the labouring samples were collected during emergency Caesarean section, performed for reasons such as breech presentation, failure to progress in labour and fetal distress. Therefore, many of the women in the study cannot be considered physiologically ‘normal’, which raises the question of whether or not the results can be generalised to a normal population. Thirdly, many of the women had undergone treatment with Syntocinon (oxytocin), prostaglandins or both to induce or augment labour. It is unclear how these treatments might effect global gene expression in the myometrium, but they are likely to contribute to the upregulation of genes with an inflammatory role. However, in a sensitivity analysis, our main results were consistent with those generated when omitting women who had undergone any pharmaceutical treatment, suggesting that pharmaceutical treatment is not a large confounding factor. This was supported by our sample-sample network graph, which suggested that labouring samples did not cluster differently according to treatment. Fourthly, tissue was collected from the upper flap of the lower transverse incision through the uterine wall, which is the most convenient location at the time of Caesarean. However, the upper segment is known to show increased contractility relative to the lower segment [14,66,67], making it arguably a more appropriate tissue to study. Furthermore, previous studies have shown significant differences in gene expression and molecular pathways associated with the initiation of labour between the upper and lower uterine segments [14,68], so the results of this study cannot necessarily be assumed to be relevant to the molecular events taking place throughout the whole organ [69]. Finally, compared to RNA-Seq, microarray technology covers less of the transcriptome. It is also less able to detect low abundance transcripts, unable to differentiate isoforms and identify genetic variants and is prone to technical issues related to probe performance [70]. However, microarray is less expensive and still currently the most commonly used technology to study the transcriptome. It is encouraging to note that there is a high degree of concordance between our findings and those generated using RNA-Seq [64].

In conclusion, a combination of statistical and network graph approaches has extended the list of genes associated with labour and provided further support that labour appears to be characterised by an apparent increase in the expression of inflammatory genes. Our study also identified an apparent decrease in the expression of muscle-specific processes in human lower segment myometrium. These findings are likely to be related to a change in the composition of the myometrial tissue at labour due to the known leukocyte infiltration at this time. Further work will be necessary to reveal whether these results are solely due to leukocyte infiltration into the myometrium as a mechanism initiating labour, or whether they represent gene changes in the myocytes themselves. Both the large sample size and the meta-analysis with previous studies improve the reliability and generalizability of our results. Appropriate analysis of high throughput studies such as this will be essential for improving our understanding of the molecular mechanisms underlying parturition, and will provide a basis for understanding differences between normal and dysfunctional labour, including PTL. In order to facilitate future work, we have made our data publically available (at www.ebi.ac.uk/arrayexpress/ under accession number E-MTAB-3136), and we would urge other researchers to do the same.

## Supporting Information

S1 File Extended meta-analysis methods. (DOCX)

S2 File Complete list of genes differentially expressed in labour. (XLSX)

S3 File MCL Clusters. (CSV)

S4 File Primary cell atlas graphs showing genes that are usually highly expressed in neutrophils. (PDF)

S5 File Supplementary Figs and tables. (DOCX)
