# Supplementary material for: Transcription Analysis of the Myometrium of Labouring and Non-Labouring Women
Source: PLoS One. 2016 May 13;11(5):e0155413. doi: 10.1371/journal.pone.0155413 (PMC4866706; doi:10.1371/journal.pone.0155413)
Supplement: S1 File — (DOCX) [file pone.0155413.s001.docx]

#### Meta-analysis Methods

#### Search strategy

A systematic approach was used to search the literature to identify all eligible studies with minimal bias. The online repositories of abstracts PubMed (http://www.pubmed.gov/), Google Scholar (http://scholar.google.com/), Web of Science (http://wos.mimas.ac.uk) and SCOPUS (http://www.scopus.com), and the microarray data repositories ArrayExpress (http://www.ebi.ac.uk/arrayexpress/) and Gene Expression Omnibus (GEO)(http://www.ncbi.nlm.nih.gov/geo) were searched using the following search terms: “myometrium” and “microarray”, “uterine smooth muscle” and “microarray”, “myometrium”, “myometrial”, “uterine smooth muscle”.

#### Inclusion/exclusion criteria

All human studies investigating myometrial gene expression in pregnant labouring or pregnant non-labouring women were eligible for inclusion in the meta-analysis. Criteria for excluding studies were as follows:

- No human samples (studies that included non-human samples were eligible only if they also included human samples).
- No myometrium samples.
- Myometrium collected from a location other than the lower uterine segment.
- No pregnant samples.
- No opportunity to compare labouring to non-labouring myometrial samples.
- Study does not report the results of a microarray experiment, or is a review.
- Samples were prepared from cells in culture rather than tissue.
- Tissue samples were cultured and/or treated before RNA extraction.
- Raw data was not available.

#### Data identification and acquisition

Ten studies were eligible for inclusion in the meta-analysis (Table S5). Raw data for two studies - Bukowski 2006 [1] (accession number E-MEXP-106) and Weiner 2010 [2] (accession number GSE9159) - were downloaded from the online repository ArrayExpress. Where studies had not made raw data available via an online repository, authors were contacted. The authors of O’Brien 2008 [3] provided normalised data on request, but raw data was not available so we did not include this study in our meta-analysis.

#### Preprocessing

Where possible, arrays were preprocessed consistently to remove any systematic differences. The Sharp 2014, Weiner 2010 and Bukowski 2006 arrays were subjected to RMA background correction before quantile normalisation and annotation using Limma (as described above). Due to the small sample size, the O’Brien 2008 array did not undergo additional preprocessing as described above. Instead, the preprocessed data (method not reported) supplied by the authors was analysed.

#### Determining background cut-off for each study

The background expression level for each study was determined by comparing the study-specific expression of certain genes (Figure SS1) to their expression in Sharp, which was used as a reference. The background level in Sharp was set at 100 (rounded from the median expression value for negative control probes), so all genes expressed at 100 +/- 1 in Sharp were considered to be “at background level” in the other arrays. The mean expression values of these genes were roughly rounded to give the background level for each array:

- Bukowski 2006 = 5
- O’Brien 2008 = 10,000
- Weiner 2010 = 150
- (Sharp=100)


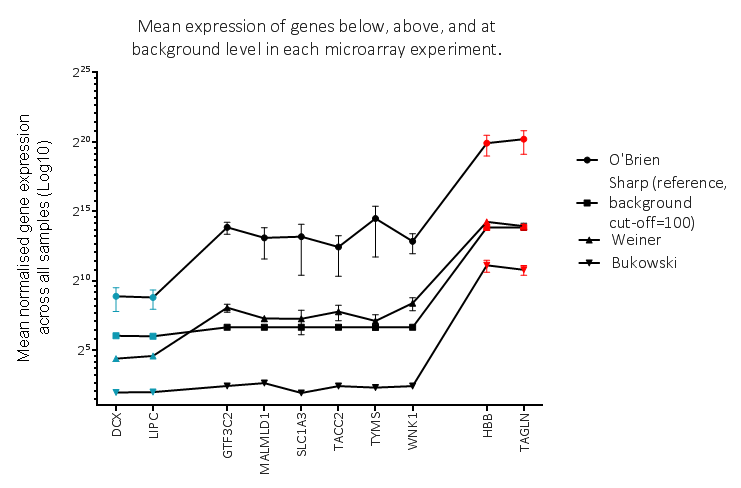


Figure SS1. Determining the background expression cut-off for each study. A plot of each study’s expression values for two genes not expected to be expressed (DCX, LIPC), six genes expected to be expressed at around background level (GTF3C2, MALMLD1, SLC1A3, TACC2, TYMS, WNK1) and two genes expected to show high expression (HBB, TAGLN), compared to their expression in Sharp 2014.

#### Statistical analysis

For each individual study, differential expression between groups was analysed as described for Sharp. We produced lists of differentially expressed genes for each study individually for a subset of agreed genes in each possible paired combination of studies (i.e. Sharp 2014 and Weiner 2010, Sharp 2014 and Bukowski 2006, Weiner 2010 and Bukowski 2006, and Sharp 2014, Weiner 2010 and Bukowski 2006). Inverse-variance-weighted meta-analysis was conducted using the R package Metafor [4] plots to compare study-specific estimates of standardised mean differences in expression values between labouring and non-labouring groups. A random effects model was used to integrate study-specific estimates and give an overall summary measure of the difference that takes study weighting into account.

1. Bukowski R, Hankins GD V, Saade GR, Anderson GD, Thornton S (2006) Labor-associated gene expression in the human uterine fundus, lower segment, and cervix. PLoS Med 3: e169. doi:10.1371/journal.pmed.0030169.

2. Weiner CPP, Mason CWW, Dong Y, Buhimschi I a A, Swaan PWW, et al. (2010) Human effector/initiator gene sets that regulate myometrial contractility during term and preterm labor. Am J Obstet Gynecol 202: 474.e1–20. doi:10.1016/j.ajog.2010.02.034.

3. O’Brien M, Morrison JJ, Smith TJ (2008) Upregulation of PSCDBP, TLR2, TWIST1, FLJ35382, EDNRB, and RGS12 gene expression in human myometrium at labor. Reprod Sci 15: 382–393. doi:10.1177/1933719108316179.

4. Viechtbauer W (2010) Conducting Meta-Analyses in R with the metafor Package. J Stat Softw 36: 1–48.
