# Supplementary figures and images for: Transcription Analysis of the Myometrium of Labouring and Non-Labouring Women
Source: PLoS One. 2016 May 13;11(5):e0155413. doi: 10.1371/journal.pone.0155413 (PMC4866706; doi:10.1371/journal.pone.0155413)

# CD48

## Probe 1

## Probe 2

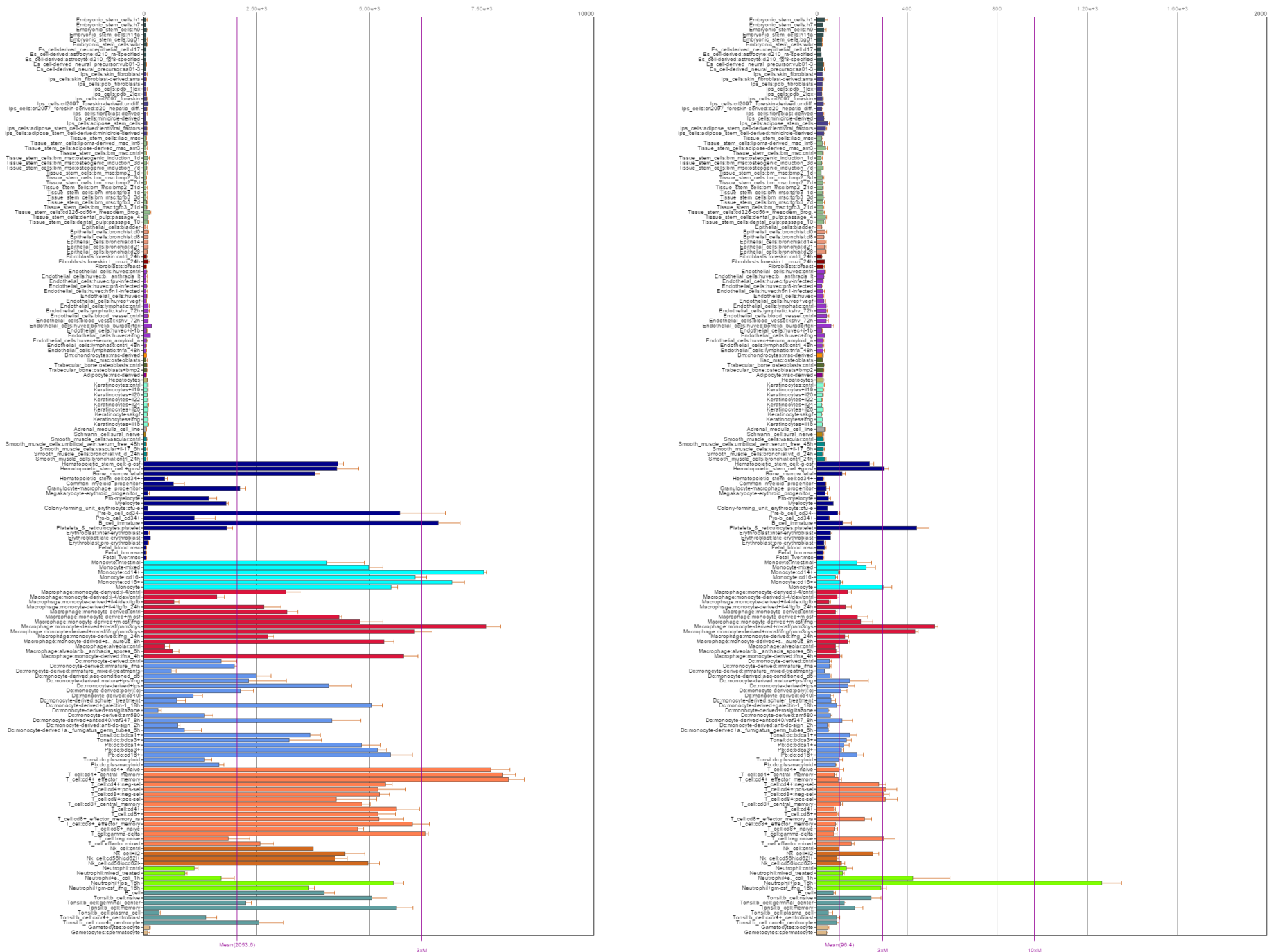

# CD53

# Probe 1

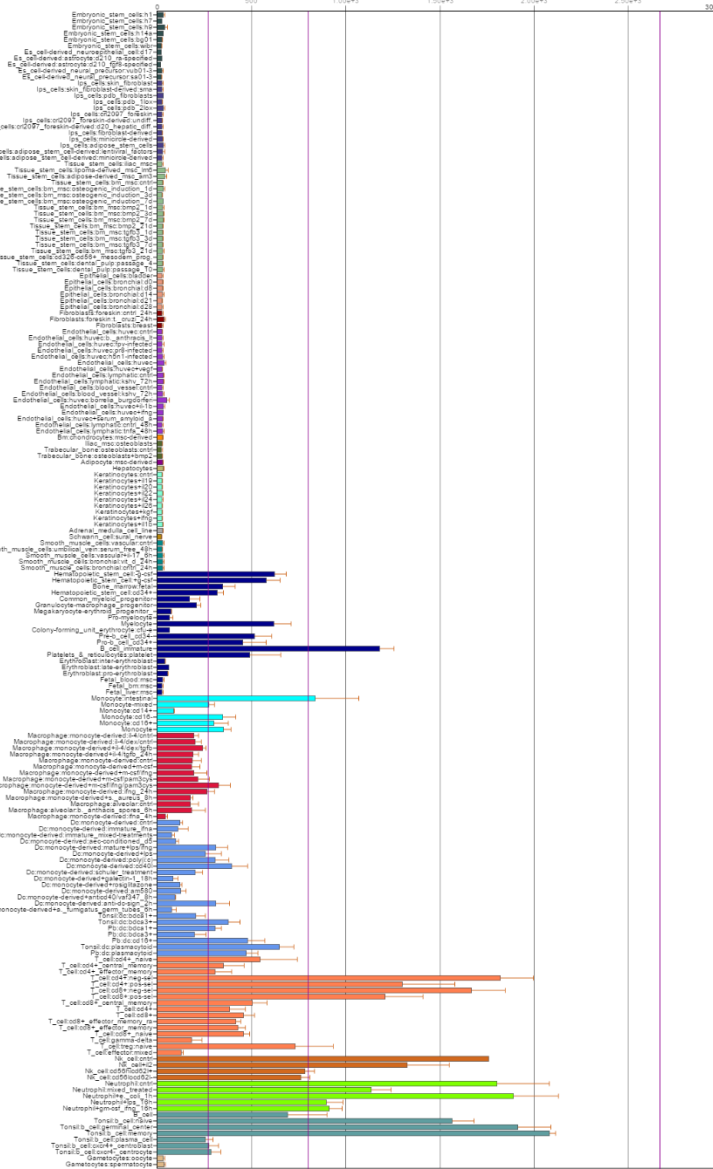

## Probe 2

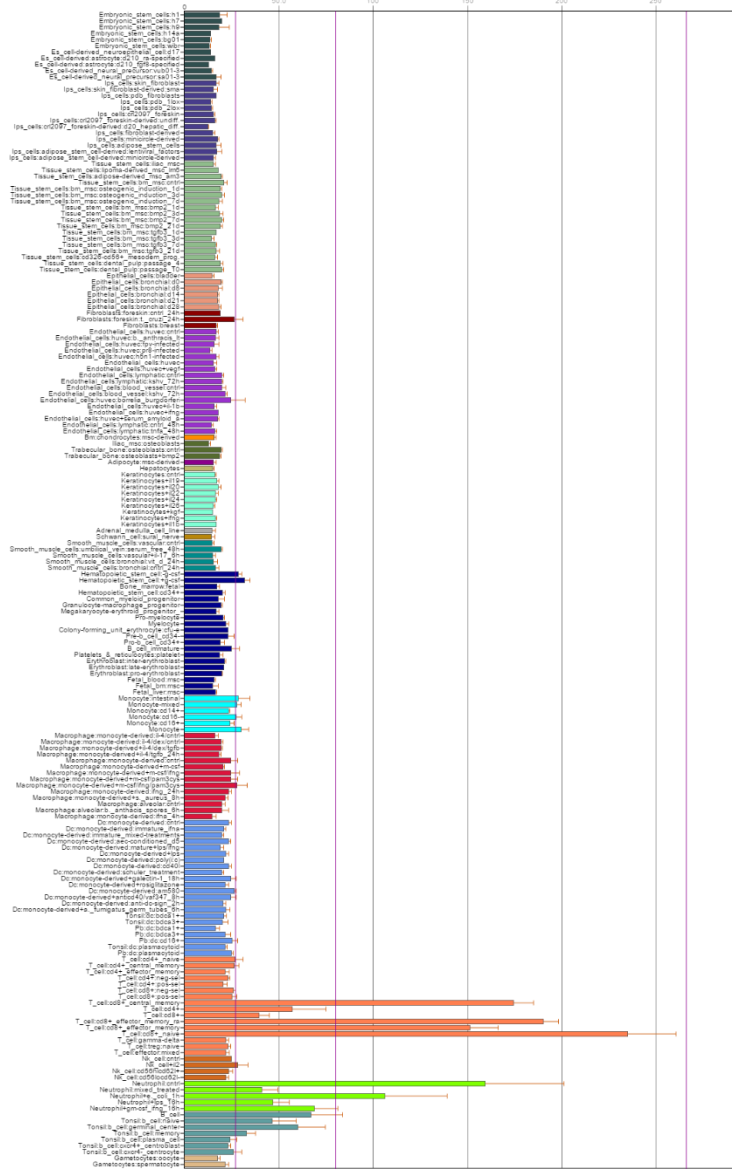

## Probe 3

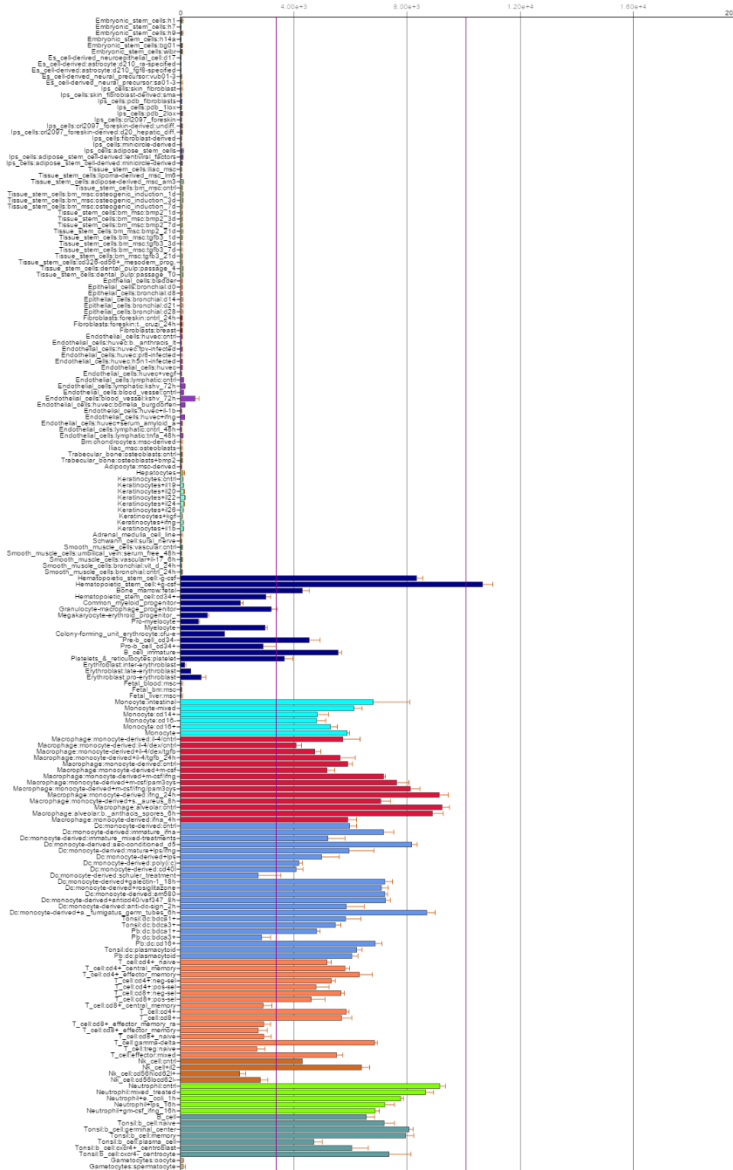

CXCL2

Probe 1

Probe 2

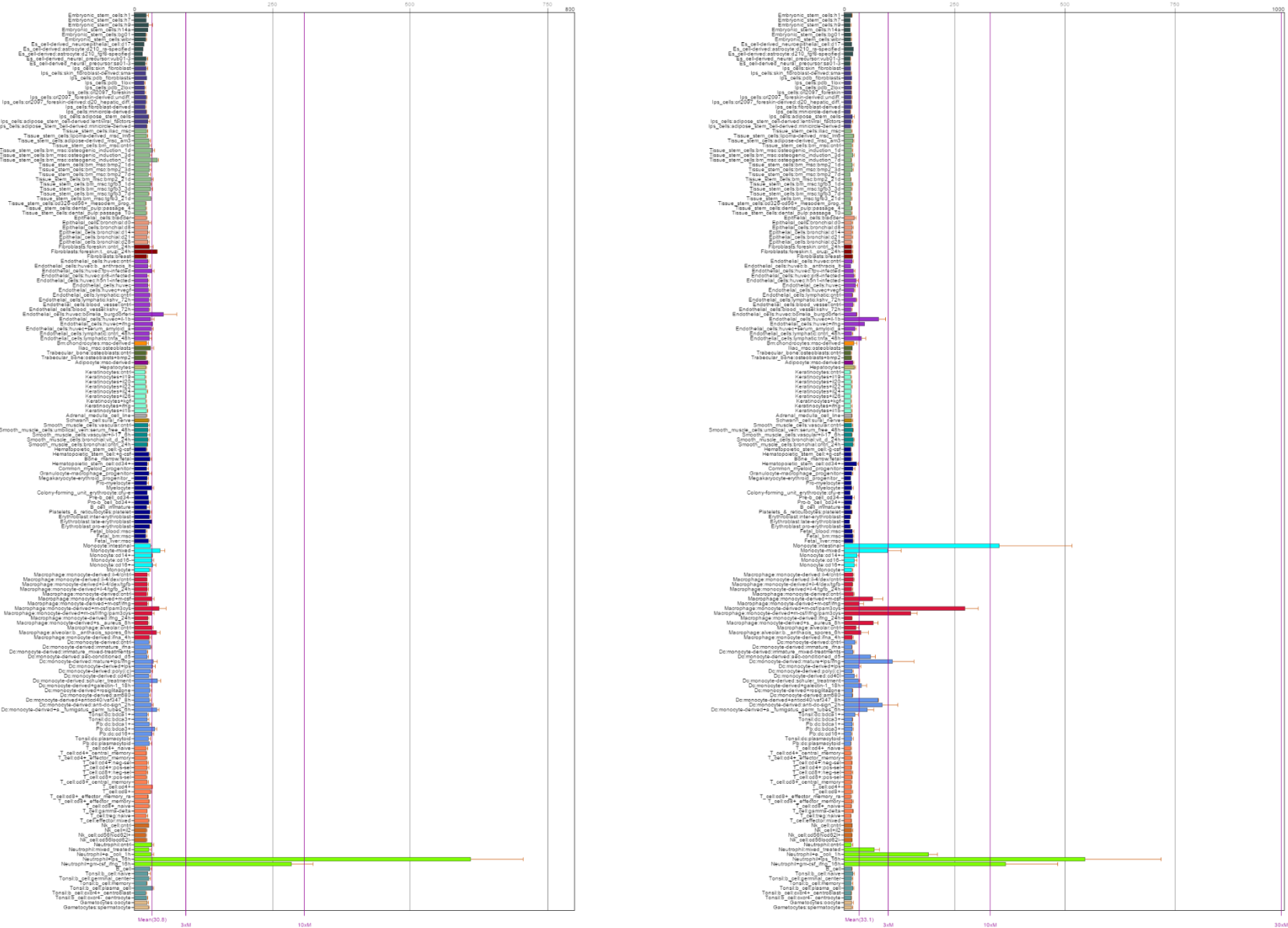

Probe 3

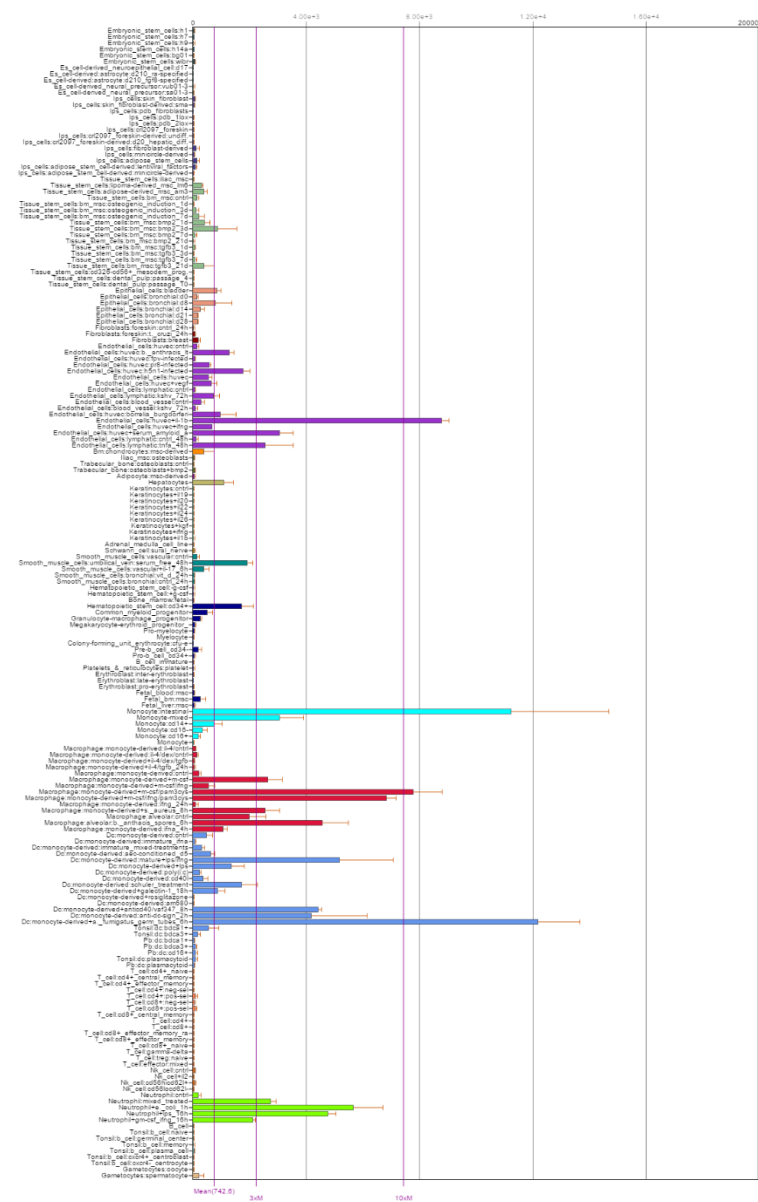

LRG1

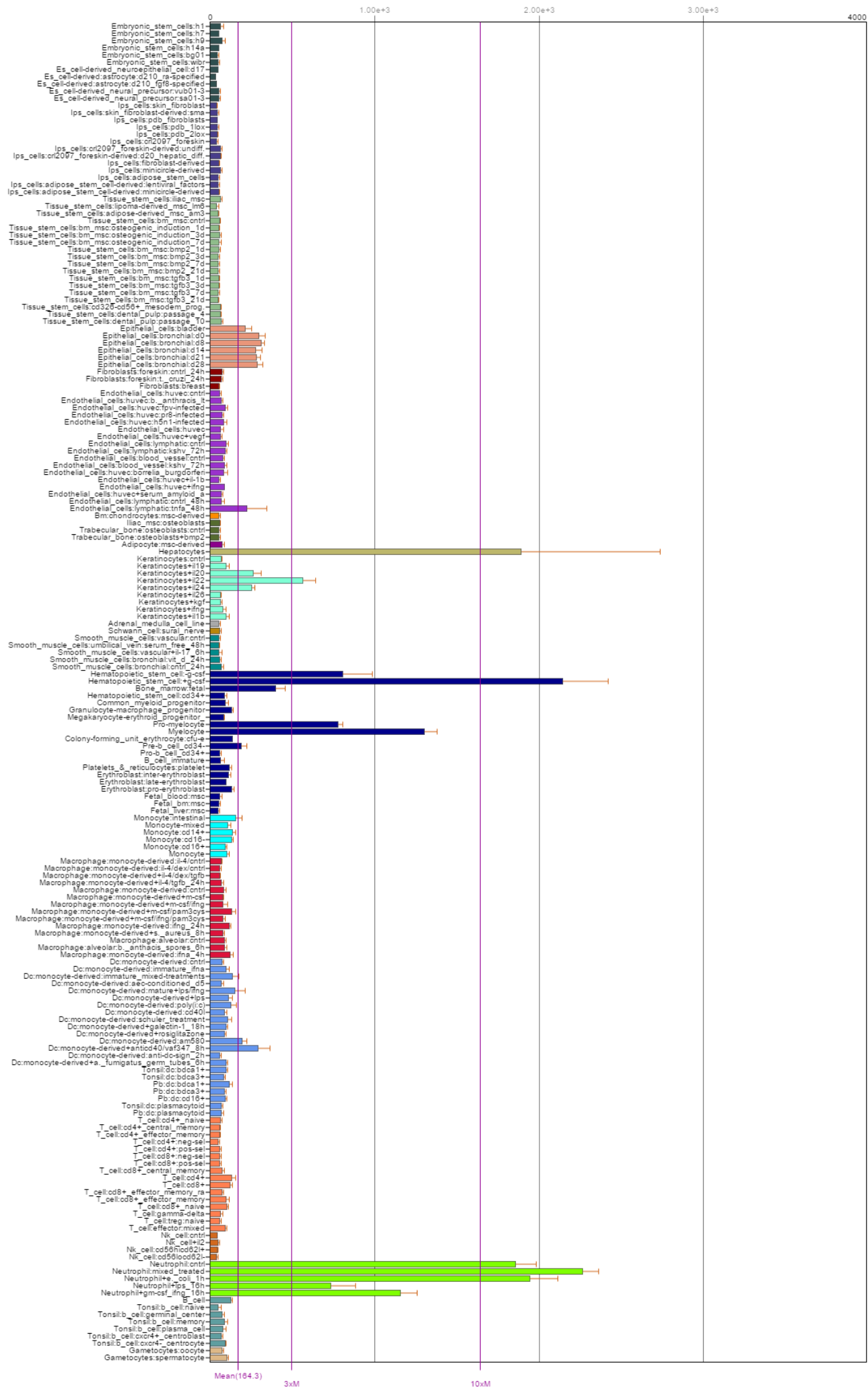

NCF2

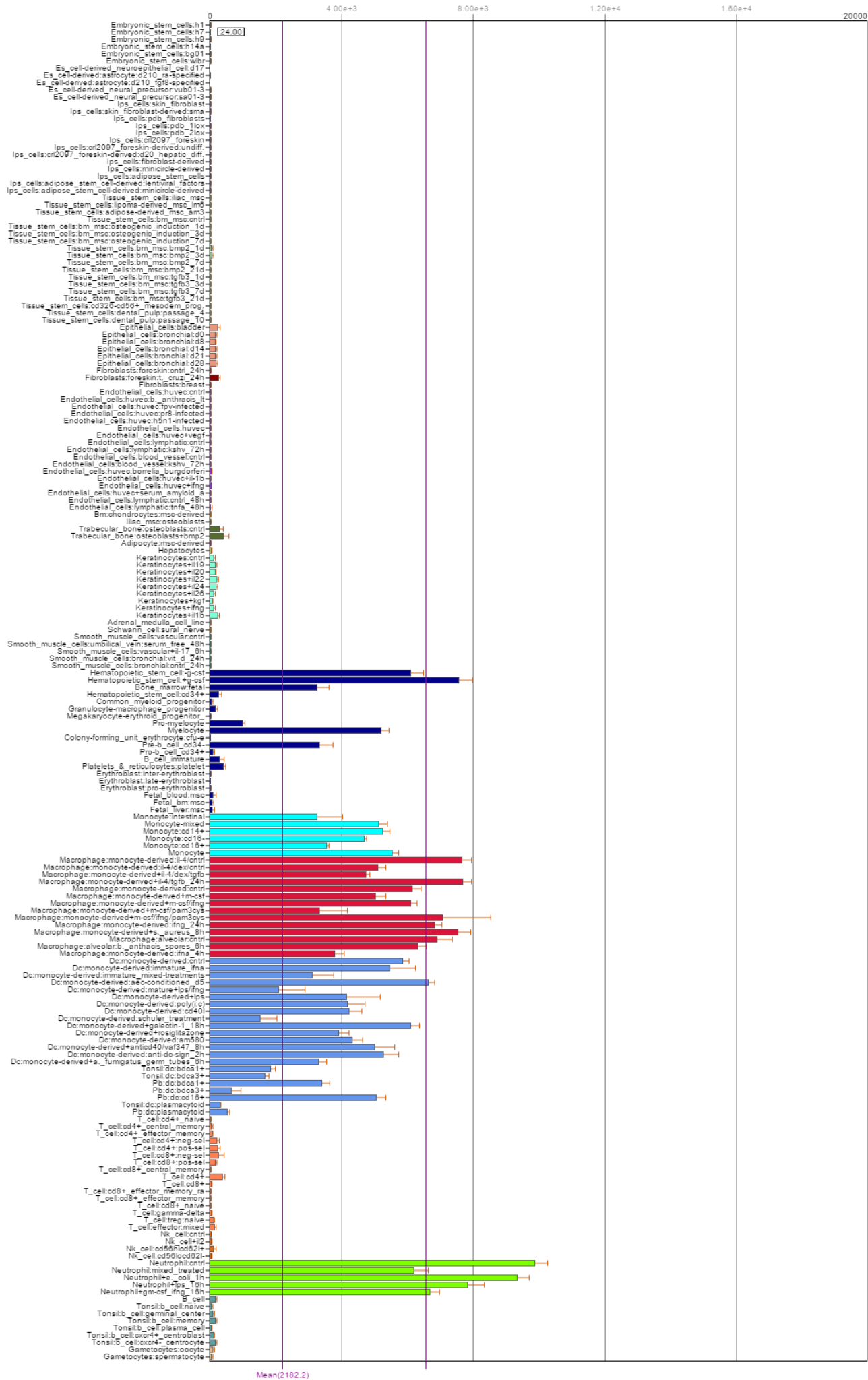

# NCF4

## Probe 1

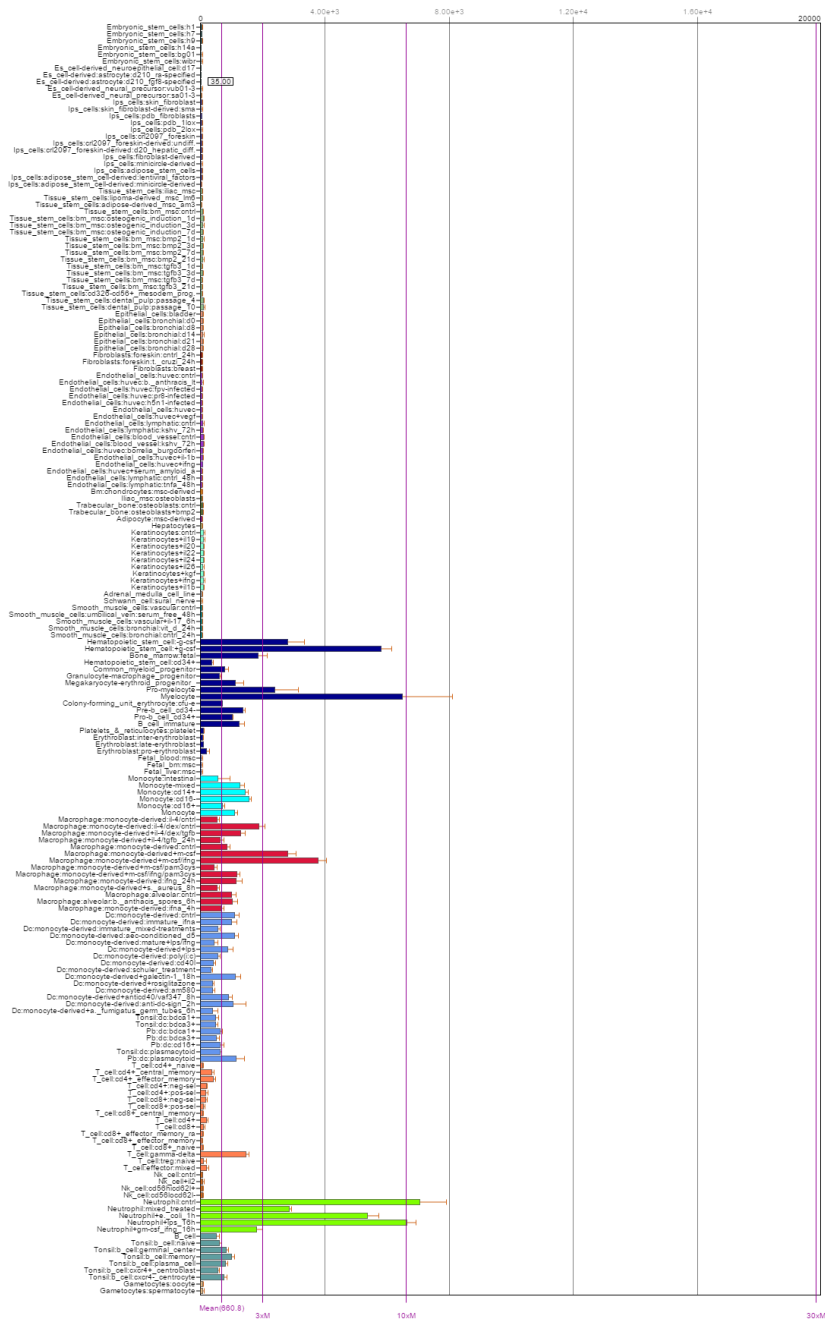

## Probe 2

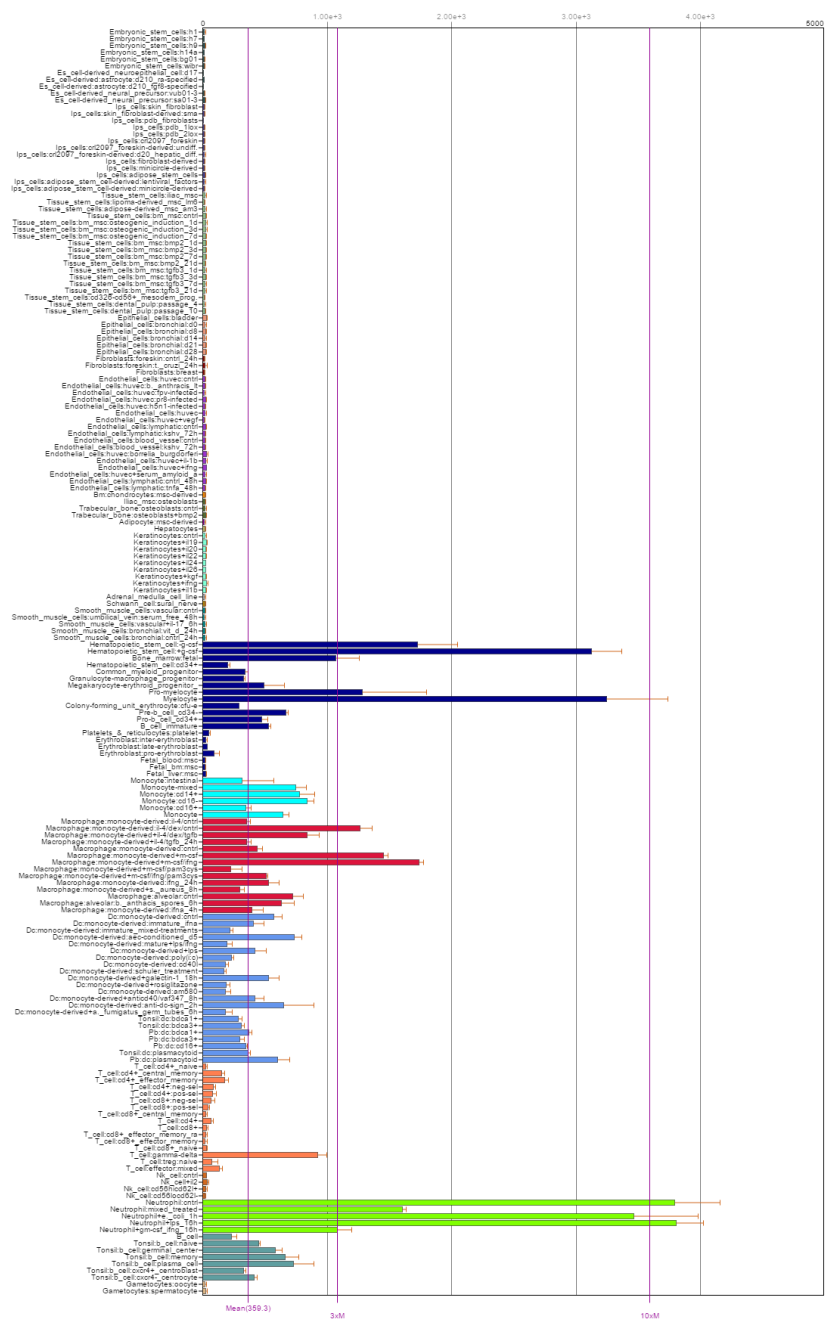

Supplement: S4 File — (PDF) [file pone.0155413.s004.pdf]
