# Supplementary material for: Transcription Analysis of the Myometrium of Labouring and Non-Labouring Women
Source: PLoS One. 2016 May 13;11(5):e0155413. doi: 10.1371/journal.pone.0155413 (PMC4866706; doi:10.1371/journal.pone.0155413)
Supplement: S5 File — (DOCX) [file pone.0155413.s005.docx]

## Supplementary Information

Table A. Information about the gene assays used to validate the microarray.

| Gene ID | Gene name | TaqMan assay ID | Reason gene chosen for validation |
| --- | --- | --- | --- |
| RBM42 | RNA binding motif protein 42 | Hs00225667_m1 | Not differentially expressed according to the array. |
| SHROOM4 | Shroom family member 4 | Hs00393349_m1 | Not differentially expressed according to the array. |
| FABP4 | Fatty acid binding protein 4 | Hs01086177_m1 | Downregulated in labour according to the array. |
| IGFBP5 | Insulin-like growth factor binding protein 2 | Hs00181213_m1 | Downregulated in labour according to the array. |
| MYH11 | Myosin heavy chain 11 | Hs00224610_m1 | Downregulated in labour according to the literature. Smooth muscle marker. |
| TPM1 | Tropomyosin 1 | Hs00165966_m1 | Downregulated in labour according to the literature. Smooth muscle marker. |
| IL6 | Interleukin 6 | Hs00985639_m1 | Upregulated in labour according to the array and the literature. |
| IL8 | Interleukin 8 | Hs00174103_m1 | Upregulated in labour according to the array and the literature. |
| MT1E | Metallothionein 1E | Hs01938284_g1 | Upregulated in labour according to the array and the literature. |
| OXTR | Oxytocin receptor | Hs00168573_m1 | Upregulated in labour according to the literature. |
| 18s (RN18s1) | 18S ribosomal RNA 1 | Hs03928985_g1 | Reference gene classically used in previous studies comparing labouring vs. non-labouring human myometrium. |

Table B. Sample characteristics

| Sample ID | Tissue storage | Indication for CS | Maternal age | BMI | Method of induction | Gestation (weeks+days) | Parity | Cervical dilation (cm) |
| --- | --- | --- | --- | --- | --- | --- | --- | --- |
| L0190 | RL | FD | 44 | 42.4 | PG & S | 40+3 | 0+2 | 9 |
| L0242 | RL | FTP | 37 | 21.6 | S | 41+3 | 0+0 | MISSING |
| L0353 | RL | MISSING | 32 | 39.8 | PG | 39+0 | 1+0 | MISSING |
| L0356 | No RL | MISSING | 21 | 43.7 | PG | 39+6 | 0+0 | MISSING |
| L0381 | No RL | MISSING | 28 | 20.7 | None | 36+4 | MISSING | MISSING |
| L2567 | RL | FD | 28 | 30.1 | None | 41+2 | 0+0 | 10 |
| L2649 | RL | FTP | 39 | 22.2 | None | 40+5 | 1+1 | 6 |
| L2688 | RL | PPROM/B | 36 | 25.8 | None | 35+0 | 1+2 | 1 |
| L3132 | RL | FTP | 32 | 22.7 | S | 40+4 | 0+0 | 4.5 |
| L3140 | RL | B | 28 | 19.1 | None | 33+4 | 0+0 | 5 |
| L3168 | RL | MISSING | 32 | 39.3 | S | 39+2 | 0+0 | 8 |
| L3171 | RL | FTP | 34 | 20.7 | None | 41+0 | 1+0 | 1 |
| L3179 | RL | FTP | 22 | 26.2 | None | 40+6 | 1+0 | 3 |
| L3189 | RL | FTP | 28 | 21.2 | S | 40+3 | 0+0 | 5 |
| L3195 | RL | B | 28 | 25.7 | None | 41+3 | 0+1 | MISSING |
| L3196 | RL | FTP | 29 | 21.6 | S | 39+6 | 0+0 | 5.6 |
| L3197 | RL | B | 39 | 21.7 | None | 40+1 | 0+0 | 2 |
| L3216 | RL | FTP | 23 | 23.1 | S | 41+4 | 0+0 | 9 |
| L3224 | RL | FTP | 33 | 27 | None | 41+6 | 0+0 | 8 |
| L3226 | RL | FD | 36 | 25.6 | None | 42+2 | 0+1 | 4 |
| L3232 | No RL | FTP | 39 | 40.3 | PG | 41+6 | 2+0 | 8 |
| L3236 | RL | FTP | 30 | 36 | S | 36+6 | MISSING | 9 |
| NL0072 | RL | MISSING | 30 | 23.9 | None | 39+0 | ≥1 | n/a |
| NL2006 | RL | B | 33 | 23.4 | None | 39+1 | 0+1 | n/a |
| NL2064 | RL | PP | 43 | 27.2 | None | 38+4 | 0+0 | n/a |
| NL2088 | RL | B | 31 | 20.5 | None | 39+5 | 0+0 | n/a |
| NL2090 | RL | O/H | 32 | 27.7 | None | 39+5 | 2+0 | n/a |
| NL2093 | RL | B | 33 | 41.2 | None | 39+0 | 1+1 | n/a |
| NL2098 | RL | B | 30 | 22 | None | 39+3 | 0+0 | n/a |
| NL2133 | RL | MISSING | 35 | 29.4 | None | 41+4 | 0+0 | n/a |
| NL2158 | RL | B | 23 | 21.1 | None | 40+1 | 0+1 | n/a |
| NL2161 | RL | O/H | 36 | 23 | None | 37+0 | 2+0 | n/a |
| NL2275 | RL | B | 38 | 23.2 | None | 39+1 | 0+0 | n/a |
| NL2285 | RL | B | 34 | 22.8 | None | 40+0 | 0+2 | n/a |
| NL2410 | RL | PP | 32 | 23.1 | None | 38+5 | 0+0 | n/a |
| NL2474 | RL | IUGR | 36 | 38.1 | None | 31+2 | 0+1 | n/a |
| NL2504 | RL | PIH | 34 | 26.6 | None | 36+4 | 1+0 | n/a |
| NL3007 | RL | B | 34 | 46.8 | None | 39+3 | 0+0 | n/a |
| NL3015 | RL | B | 40 | 26.5 | None | 39+2 | 0+1 | n/a |
| NL3087 | RL | PCS | 31 | 43.0 | None | 39+4 | 1+2 | n/a |
| NL3095 | RL | PCS | 26 | 26.3 | None | 39+1 | 1+1 | n/a |
| NL3114 | RL | PCS | 33 | 32.3 | None | 39+2 | 1+0 | n/a |
| NL3127 | RL | B | 31 | 31.7 | None | 39+3 | 0+0 | n/a |
| NL3133 | RL | MISSING | 35 | 44.4 | None | 39+4 | 0+0 | n/a |
| NL3142 | RL | MISSING | 38 | 24.6 | None | 39+5 | 1+0 | n/a |
| NL3148 | RL | Hip | 22 | 24.3 | None | 39+2 | 0+0 | n/a |
| NL318 | RL | B | 29 | 23.9 | None | 39+5 | 2+0 | n/a |
| NL3247 | RL | O/H | 32 | 22.2 | None | 39+0 | 1+0 | n/a |

RL = RNA later, FD = Fetal distress, FTP = Failure to progress, MISSING = Not recorded, PPROM = Preterm prelabour rupture of membranes, B = Breech, PP = Placenta praevia, O/H = Obstetric history, IUGR = intrauterine growth restriction, PIH = Pregnancy-induced hypertension, PCS = Previous Caesarean section, Hip = Hip problems, PG = Prostaglandin, S = Syntocinon (oxytocin)

Table C. The top five clusters of GO terms associated with genes that show higher expression in labouring samples than non-labouring samples.

| DAVID cluster | Cluster enrichment score | GO Term | Genes linked to this term | % of total genes in the list (923) | P Value |
| --- | --- | --- | --- | --- | --- |
| 1 | 15.07 | GO:0006952~defense response | 91 | 9.86 | 7E-18 |
|  |  | GO:0009611~response to wounding | 78 | 8.45 | 4E-15 |
|  |  | GO:0006954~inflammatory response | 57 | 6.18 | 2E-14 |
| 2 | 8.5 | GO:0042330~taxis | 35 | 3.79 | 8E-12 |
|  |  | GO:0006935~chemotaxis | 35 | 3.79 | 8E-12 |
|  |  | GO:0007626~locomotory behavior | 38 | 4.12 | 5E-07 |
|  |  | GO:0007610~behavior | 52 | 5.63 | 3E-06 |
| 3 | 5.87 | GO:0010033~response to organic substance | 96 | 10.40 | 1E-15 |
|  |  | GO:0048545~response to steroid hormone stimulus | 28 | 3.03 | 8E-06 |
|  |  | GO:0009719~response to endogenous stimulus | 41 | 4.44 | 3E-04 |
|  |  | GO:0009725~response to hormone stimulus | 38 | 4.12 | 3E-04 |
|  |  | GO:0043627~response to estrogen stimulus | 14 | 1.52 | 5E-03 |
| 4 | 5.16 | GO:0043066~negative regulation of apoptosis | 51 | 5.53 | 9E-10 |
|  |  | GO:0043069~negative regulation of programmed cell death | 51 | 5.53 | 2E-09 |
|  |  | GO:0060548~negative regulation of cell death | 51 | 5.53 | 2E-09 |
|  |  | GO:0042981~regulation of apoptosis | 81 | 8.78 | 2E-07 |
|  |  | GO:0043067~regulation of programmed cell death | 81 | 8.78 | 3E-07 |
|  |  | GO:0010941~regulation of cell death | 81 | 8.78 | 3E-07 |
|  |  | GO:0006916~anti-apoptosis | 31 | 3.36 | 1E-06 |
|  |  | GO:0043065~positive regulation of apoptosis | 38 | 4.12 | 5E-03 |
|  |  | GO:0043068~positive regulation of programmed cell death | 38 | 4.12 | 6E-03 |
|  |  | GO:0010942~positive regulation of cell death | 38 | 4.12 | 6E-03 |
|  |  | GO:0006917~induction of apoptosis | 27 | 2.93 | 3E-02 |
|  |  | GO:0012502~induction of programmed cell death | 27 | 2.93 | 3E-02 |
| 5 | 4.92 | GO:0046651~lymphocyte proliferation | 13 | 1.41 | 2E-06 |
|  |  | GO:0070661~leukocyte proliferation | 13 | 1.41 | 3E-06 |
|  |  | GO:0032943~mononuclear cell proliferation | 13 | 1.41 | 3E-06 |
|  |  | GO:0045321~leukocyte activation | 33 | 3.58 | 4E-06 |
|  |  | GO:0046649~lymphocyte activation | 29 | 3.14 | 5E-06 |
|  |  | GO:0001775~cell activation | 36 | 3.90 | 1E-05 |
|  |  | GO:0042110~T cell activation | 19 | 2.06 | 2E-04 |
|  |  | GO:0042098~T cell proliferation | 8 | 0.87 | 5E-04 |

Table D. The top five clusters of GO terms associated with genes that show lower expression in labouring samples than non-labouring samples.

| DAVID cluster | Cluster enrichment score | GO Term | Genes linked to this term | % of total genes in the list (749) | P Value |
| --- | --- | --- | --- | --- | --- |
| 1 | 4.24 | GO:0048545~response to steroid hormone stimulus | 26 | 3.47 | 2.5E-07 |
|  |  | GO:0031960~response to corticosteroid stimulus | 12 | 1.60 | 6.2E-04 |
|  |  | GO:0051384~response to glucocorticoid stimulus | 11 | 1.47 | 1.2E-03 |
| 2 | 4.09 | GO:0009725~response to hormone stimulus | 40 | 5.34 | 3.7E-08 |
|  |  | GO:0009719~response to endogenous stimulus | 41 | 5.47 | 1.8E-07 |
|  |  | GO:0048545~response to steroid hormone stimulus | 26 | 3.47 | 2.5E-07 |
|  |  | GO:0043627~response to estrogen stimulus | 17 | 2.27 | 4.8E-06 |
|  |  | GO:0010033~response to organic substance | 55 | 7.34 | 9.3E-06 |
|  |  | GO:0032355~response to estradiol stimulus | 11 | 1.47 | 5.3E-05 |
|  |  | GO:0032870~cellular response to hormone stimulus | 16 | 2.14 | 3.2E-04 |
|  |  | GO:0043434~response to peptide hormone stimulus | 16 | 2.14 | 1.5E-03 |
|  |  | GO:0032868~response to insulin stimulus | 9 | 1.20 | 5.0E-02 |
|  |  | GO:0032869~cellular response to insulin stimulus | 7 | 0.93 | 5.7E-02 |
|  |  | GO:0008286~insulin receptor signaling pathway | 4 | 0.53 | 1.9E-01 |
| 3 | 3.67 | GO:0007517~muscle organ development | 26 | 3.47 | 1.5E-06 |
|  |  | GO:0014706~striated muscle tissue development | 15 | 2.00 | 3.3E-04 |
|  |  | GO:0060537~muscle tissue development | 15 | 2.00 | 5.4E-04 |
|  |  | GO:0007519~skeletal muscle tissue development | 10 | 1.34 | 1.3E-03 |
|  |  | GO:0060538~skeletal muscle organ development | 10 | 1.34 | 1.3E-03 |
| 4 | 3.67 | GO:0007517~muscle organ development | 26 | 3.47 | 1.5E-06 |
|  |  | GO:0014706~striated muscle tissue development | 15 | 2.00 | 3.3E-04 |
|  |  | GO:0060537~muscle tissue development | 15 | 2.00 | 5.4E-04 |
|  |  | GO:0007519~skeletal muscle tissue development | 10 | 1.34 | 1.3E-03 |
|  |  | GO:0060538~skeletal muscle organ development | 10 | 1.34 | 1.3E-03 |
| 5 | 3.67 | GO:0007010~cytoskeleton organization | 37 | 4.94 | 4.2E-05 |
|  |  | GO:0030036~actin cytoskeleton organization | 24 | 3.20 | 4.8E-05 |
|  |  | GO:0030029~actin filament-based process | 24 | 3.20 | 1.3E-04 |
|  |  | GO:0007015~actin filament organization | 9 | 1.20 | 8.5E-03 |

Table E. Studies eligible for the meta-analysis.

| First author | Microarray platform | Groups compared (number of LS myometrial samples) | Inclusion in meta-analysis |
| --- | --- | --- | --- |
| Aguan (2000)[1] | Atlas Human cDNA array blots | TNIL (3)  TIL (3) | No reply from authors |
| Bethin (2003)[2] | Affymetrix Human U95A | PTNIL (3)  PTIL (3)  TIL (3) | No reply from authors |
| Charpigny (2003)[3] | Atlas Human 1.2 and 1.2II nylon cDNA expression macroarrays | PTNIL (4)  TNIL (4)  TIL (4) | Little information available regarding unusual platform |
| Havelock (2005)[4] | Incyte Human UniGEM V .14 | NIL (6)  TIL (4) | No reply from authors |
| Bukowski (2006)[5] | Affymetrix Human U95A | TNIL (6)  TIL (7) | Raw data available in online repository |
| Esplin (2005)[6] | Unknown (cDNA hybridized to two glass chips and imaged using the Molecular Dynamics Generation III scanner) | TNIL (5)  TIL (5) | Little information available regarding unusual platform |
| O'Brien (2008)[7] | Applied 130 Biosystems Genome Survey Microarray (version 2) | TNIL (3)  TIL (3) | Original raw data not available |
| Bollapragada (2009)[8] | Affymetrix Human U133 + 2.0 | TNIL (9)  TIL (9) | Original raw data not available |
| Mittal (2010)[9] | Illumina Human HT-12 Expression Bead-chip | TNIL (20)  TIL (19) | Authors declined to supply data |
| Weiner (2010)[10] | Affymetrix Human U133 + 2.0 | PTNIL (3)  PTIL (3)  TNIL (3)  TIL (3) | Raw data available in online repository |

TNIL, Term not in labour; TIL, Term in labour; PTNIL, Preterm not in labour; PTIL, Preterm in labour; NIL, Not in labour; LS, Lower segment.

1. Aguan K, Carvajal J (2000) Application of a functional genomics approach to identify differentially expressed genes in human myometrium during pregnancy and labour. Mol Hum … 6: 1141–1145.

2. Bethin KE, Nagai Y, Sladek R, Asada M, Sadovsky Y, et al. (2003) Microarray analysis of uterine gene expression in mouse and human pregnancy. Mol Endocrinol 17: 1454–1469. doi:10.1210/me.2003-0007.

3. Charpigny G, Leroy M-J, Breuiller-Fouché M, Tanfin Z, Mhaouty-Kodja S, et al. (2003) A functional genomic study to identify differential gene expression in the preterm and term human myometrium. Biol Reprod 68: 2289–2296. doi:10.1095/biolreprod.102.013763.

4. Havelock JC, Keller P, Muleba N, Mayhew BA, Casey BM, et al. (2005) Human myometrial gene expression before and during parturition. Biol Reprod 72: 707–719. doi:10.1095/biolreprod.104.032979.

5. Bukowski R, Hankins GD V, Saade GR, Anderson GD, Thornton S (2006) Labor-associated gene expression in the human uterine fundus, lower segment, and cervix. PLoS Med 3: e169. doi:10.1371/journal.pmed.0030169.

6. Esplin MS, Fausett MB, Peltier MR, Hamblin S, Silver RM, et al. (2005) The use of cDNA microarray to identify differentially expressed labor-associated genes within the human myometrium during labor. Am J Obstet Gynecol 193: 404–413. doi:10.1016/j.ajog.2004.12.021.

7. O’Brien M, Morrison JJ, Smith TJ (2008) Upregulation of PSCDBP, TLR2, TWIST1, FLJ35382, EDNRB, and RGS12 gene expression in human myometrium at labor. Reprod Sci 15: 382–393. doi:10.1177/1933719108316179.

8. Bollapragada S, Bollopragada S, Youssef R, Jordan F, Greer I, et al. (2009) Term labor is associated with a core inflammatory response in human fetal membranes, myometrium, and cervix. Am J Obstet Gynecol 200: 104.e1–11. doi:10.1016/j.ajog.2008.08.032.

9. Mittal P, Romero R, Tarca AL, Gonzalez J, Draghici S, et al. (2010) Characterization of the myometrial transcriptome and biological pathways of spontaneous human labor at term. J Perinat Med 38: 617–643. doi:10.1515/JPM.2010.097.

10. Weiner CPP, Mason CWW, Dong Y, Buhimschi I a A, Swaan PWW, et al. (2010) Human effector/initiator gene sets that regulate myometrial contractility during term and preterm labor. Am J Obstet Gynecol 202: 474.e1–20. doi:10.1016/j.ajog.2010.02.034.

Table F. Heterogeneity statistics from inverse-variance meta-analysis.

|  | tau2 | P |
| --- | --- | --- |
| IL6 | 1.3715 | 0.01 |
| CXCL2 | 0.7618 | 0.02 |
| MT1E | 0.5367 | 0.04 |
| AHNAK | 0.7296 | 0.04 |
| MYH11 | 0.4821 | 0.05 |
| CD48 | 0.5572 | 0.06 |
| SCARA3 | 0.7669 | 0.06 |
| MYL9 | 0.4244 | 0.06 |
| CD53 | 0.4496 | 0.07 |
| IL8 | 0.2301 | 0.16 |
| TAGLN | 0.2323 | 0.16 |
| TPM1 | 0.1778 | 0.21 |
| AHNAK2 | 0.0000 | 0.23 |
| PLCL1 | 0.1077 | 0.28 |
| NCF2 | 0.1101 | 0.31 |
| RAB11FIP2 | 0.0190 | 0.43 |
| TMEM123 | 0.0000 | 0.44 |
| RBM42 | 0.0000 | 0.55 |
| NCF4 | 0.0000 | 0.56 |
| OXTR | 0.0000 | 0.57 |
| FABP4 | 0.0000 | 0.58 |
| LRG1 | 0.0000 | 0.74 |
| SHROOM4 | 0.0000 | 0.80 |
| IGFBP5 | 0.0000 | 0.97 |


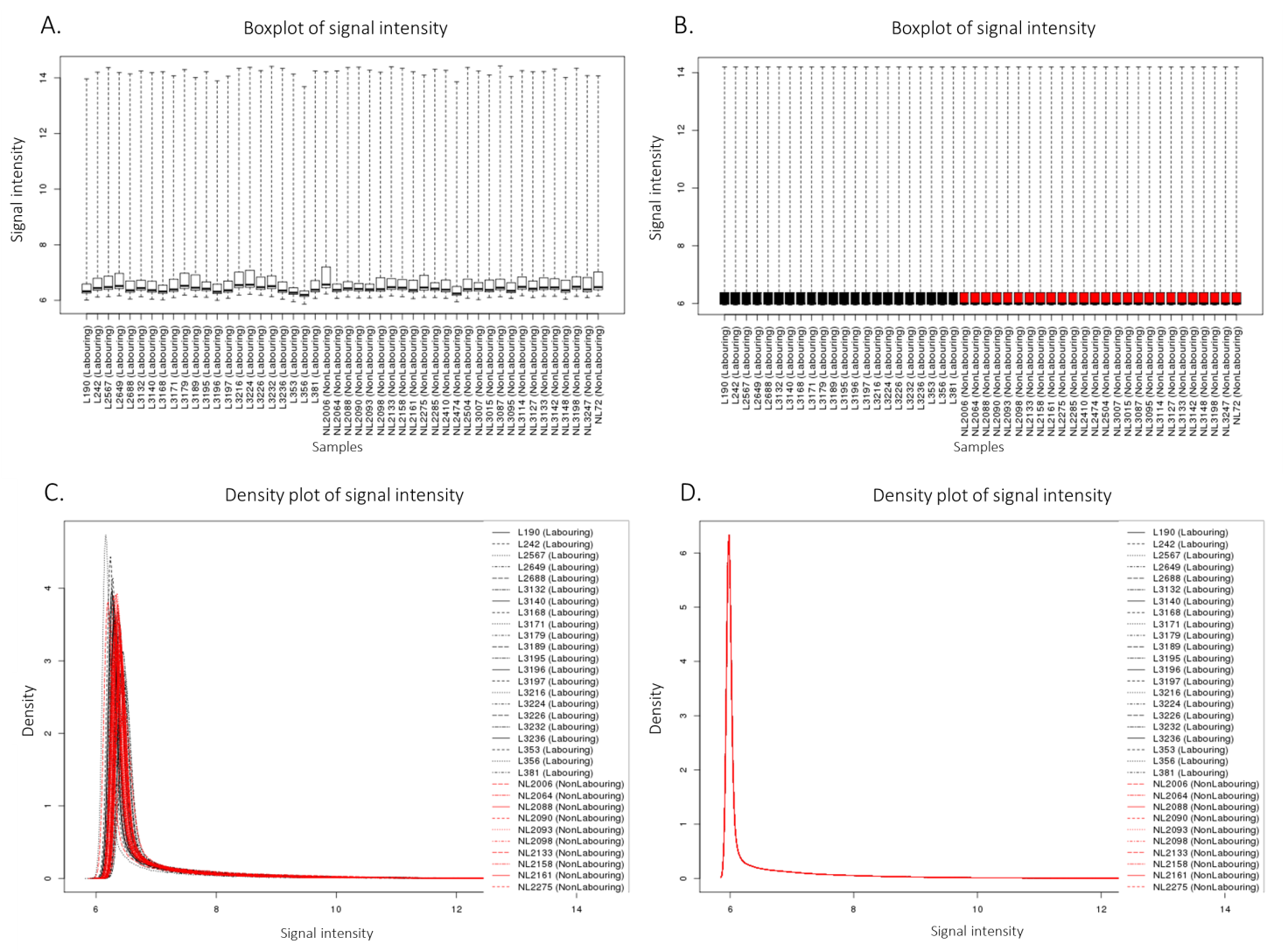


Figure A. Boxplots and density plots of signal intensity (expression value) before (A, C) and after (B,D) quantile normalisation.


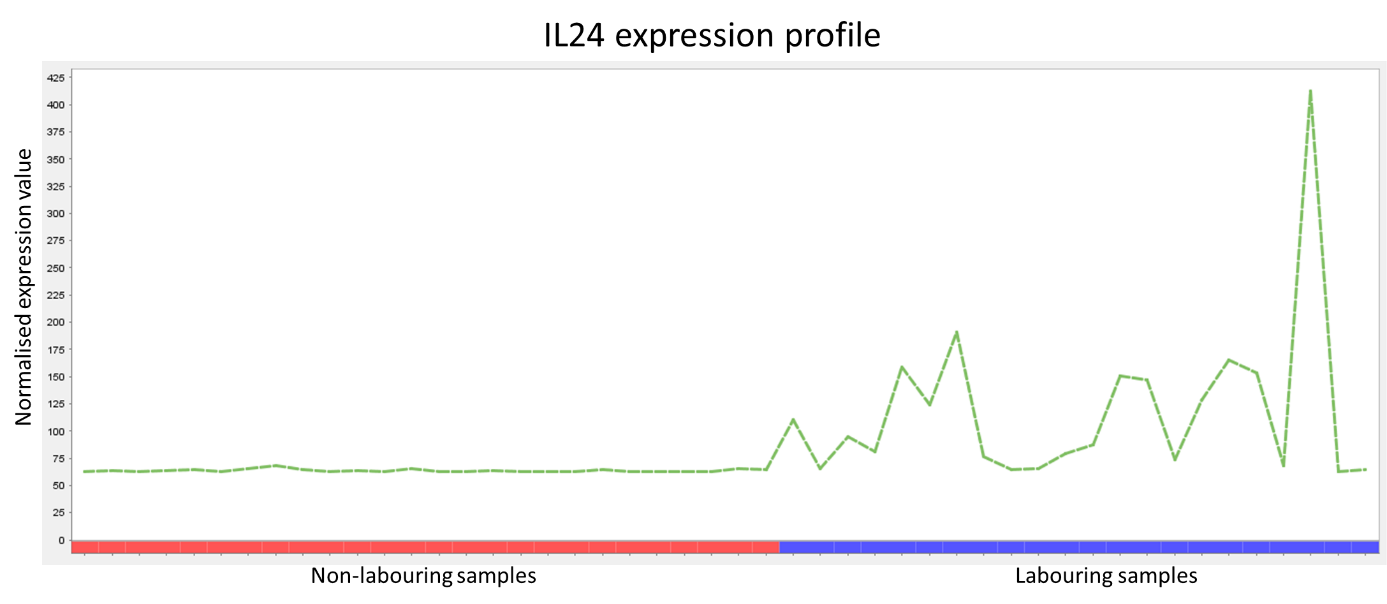


Figure B. Expression profile of IL24.


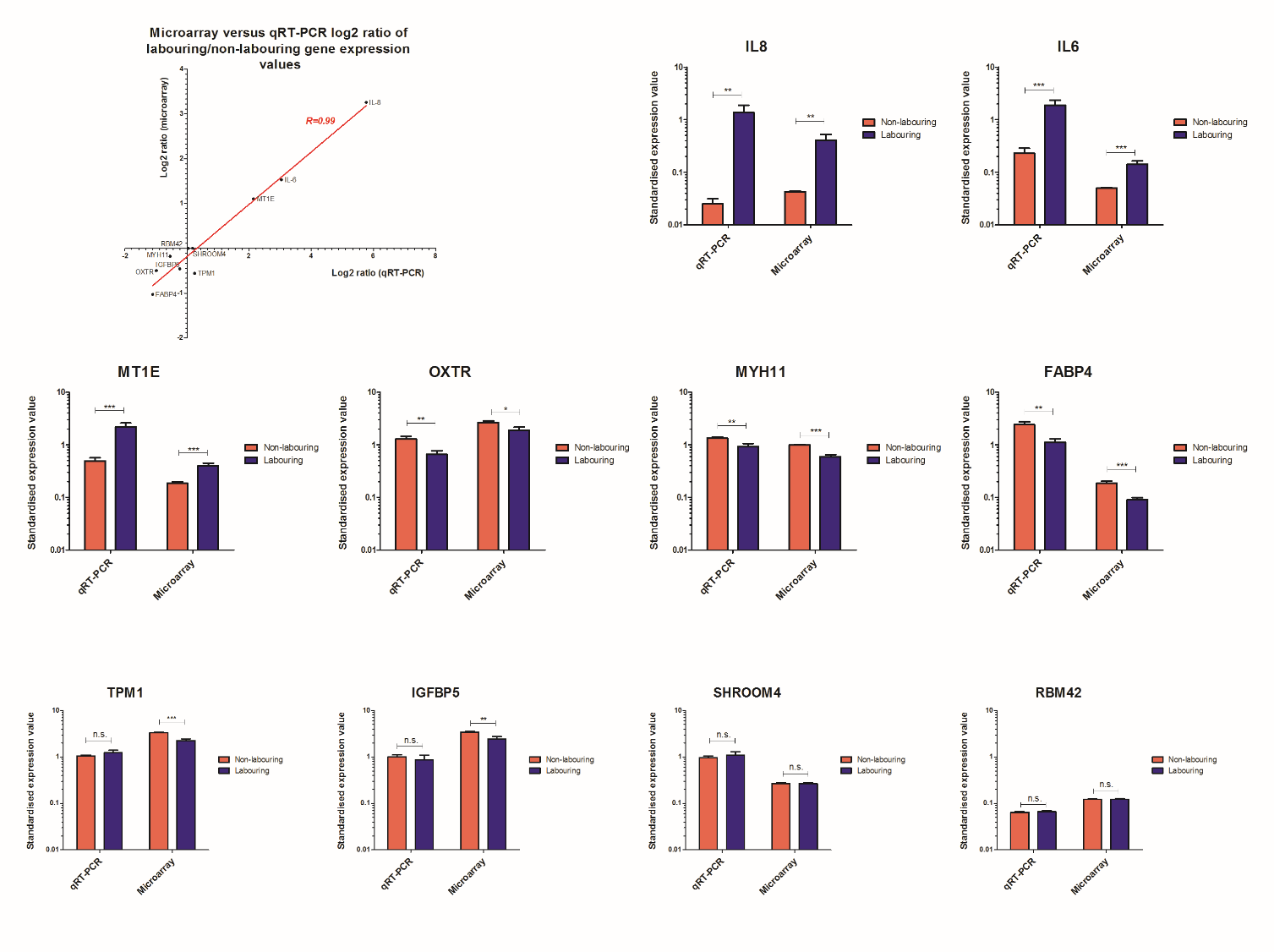


Figure C. qRT-PCR validation of microarray results. The scatter plot shows how log2 ratios of non-labouring to labouring groups correlate between technologies. Bar charts are based on normalised qRT-PCdelta CT values and normalised microarray expression values, standardised by dividing the value for each sample by the mean for all samples. The height of the bar indicates the mean standardised expression value and error bars indicate the standard error of the mean. Stars indicate the level of significance according to t-tests of delta CT values or microarray expression values.

Fold changes and 95% confidence intervals:

| Genes | PCR | Microarray |
| --- | --- | --- |
| IL8 | 54.54 (13.61, 135.22) | 9.55 (3.97, 15.29) |
| IL6 | 8.20 (3.77, 17.66) | 2.89 (2.05, 3.76) |
| MT1E | 4.41 (2.63, 7.13) | 2.15 (1.54, 2.82) |
| OXTR | -1.98 (-3.18, -1.33) | -1.41 (-2.10, -1.03) |
| MYH11 | -1.45 (-2.14, -1.07) | -1.67 (-2.07, -1.39) |
| FABP4 | -2.15 (-3.42, -1.38) | -2.04 (-2.63, -1.56) |
| TPM1 | 1.18 (-1.21, 1.58) | -1.47 (-1.79, -1.24) |
| IGFBP5 | -1.18 (-2.69, 1.40) | -1.37 (-1.83, -1.08) |
| SHROOM4 | 1.13 (-1.39, 1.60) | 1.00 (-1.11, 1.11) |
| RBM42 | 1.03 (-1.19, 1.23) | -1.00 (-1.10, 1.09) |


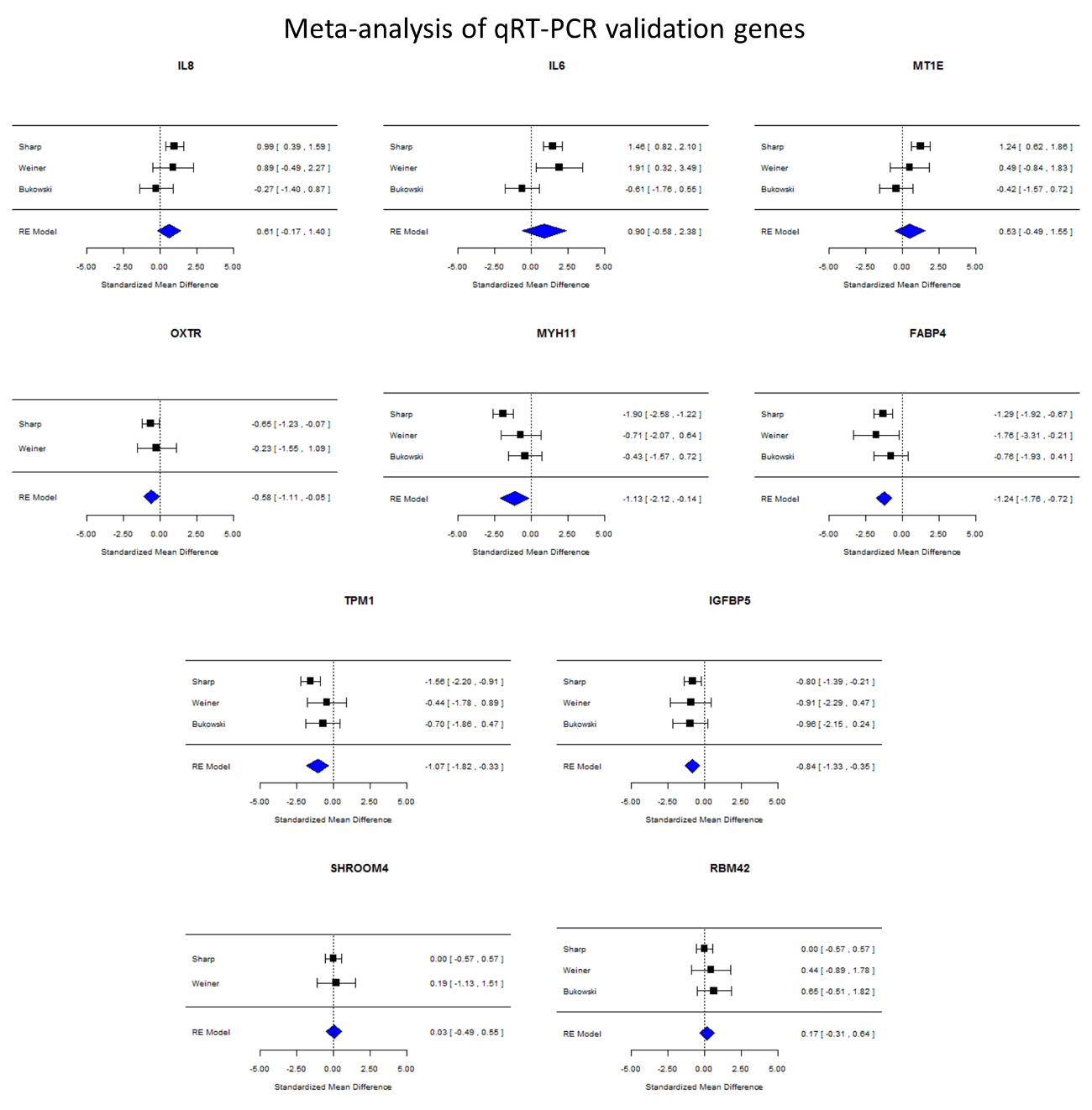


Figure D. Forest plots of standardised mean differences between labouring and non-labouring samples in Sharp, Weiner and Bukowski for the ten genes selected for qRT-PCR validation. Summary statistics, indicated by the blue diamond, were calculated via inverse variance meta-analysis. The array platform used by Bukowski did not cover all ten genes, so Bukowski could not be included in all meta-analyses.
